# Blockchain for sustainable supply chain management: trends and ways forward

**DOI:** 10.1007/s10660-022-09569-1

**Published:** 2022-05-27

**Authors:** Saumyaranjan Sahoo, Satish Kumar, Uthayasankar Sivarajah, Weng Marc Lim, J. Christopher Westland, Ashwani Kumar

**Affiliations:** 1 Jaipuria Institute of Management, Jaipur, Rajasthan India; 2 Department of Management Studies, Malaviya National Institute of Technology Jaipur, Jaipur, Rajasthan 302017 India; 3 School of Management, Faculty of Management, Law and Social Sciences, University of Bradford, Richmond Road, Bradford, BD7 1DP UK; 4 School of Business, Law and Entrepreneurship, Swinburne University of Technology, John Street, 3122, Hawthorn, Victoria Australia; 5 Faculty of Business, Design and Arts, Swinburne University of Technology, Jalan Simpang Tiga, 93350 Kuching, Sarawak Malaysia; 6 Department of Information and Decision Sciences, University of Illinois – Chicago, 601, S. Morgan Street, Chicago, IL 60607-7124 USA; 7 Indian Institute of Management, Rohtak, Haryana India

**Keywords:** Bibliometric analysis, Blockchain, Supply chain, Sustainability, Systematic literature review

## Abstract

Blockchain operates on a highly secured framework, and its decentralized consensus has benefits for supply chain sustainability. Scholars have recognized the growing importance of sustainability in supply chains and studied the potential of blockchain for sustainable supply chain management. However, no study has taken stock of high-quality research in this area. To address this gap, this paper aims to provide a state-of-the-art overview of high-quality research on blockchain for sustainable supply chain management. To do so, this paper conducts a systematic literature review using a bibliometric analysis of 146 high-quality articles on blockchain for sustainable supply chain management that have been published in journals ranked “A*”, “A”, and “B” by the Australian Business Deans Council and retrieved from the Scopus database. In doing so, this paper unpacks the most prominent journals, authors, institutions, and countries that have contributed to three major themes in the field, namely blockchain for sustainable business activities, decision support systems using blockchain, and blockchain for intelligent transportation system. This paper also reveals the use of blockchain for sustainable supply chain management across four major sectors, namely food, healthcare, manufacturing, and infrastructure, and concludes with suggestions for future research in each sector.

## Introduction

A supply chain (SC) is an essential aspect of every business that consists of downstream and upstream activities between various organizational stakeholders across functional verticals, generating value through the effective and efficient delivery of products (e.g., goods, services) [1, 2]. However, SC activities can produce not just desired but also undesired consequences [3, 4], raising concerns about its sustainability on a range of economic, environmental, regulatory, and social issues [5, 6]. Furthermore, customers may lose trust in a firm and stop investing in it if they feel that the firm has not kept itself sufficiently accountable to the preservation of cultural diversity, the environment, and other societal expectations [2, 3, 7]. In this regard, firms are increasingly engaging in sustainability practices and demanding that their SC partners practice the same in order to satisfy their social responsibility and sustain their competitiveness in the marketplace [4, 8, 9].

The idea and practice of sustainable SC management (SSCM) has gained significant traction, with numerous frameworks developed, introduced, and implemented across various sectors [10–13]. In essence, SSCM is characterized by the triple bottom line (TBL) model that aims to accomplish an equilibrium between environmental protection (planet), social responsibility (people), and economic prosperity (profit) while managing the SC [5, 14, 15]. The agreement among SC partners and their commitment that raw materials, products, procedures, and practices within the SC adhere to sustainability requirements and regulatory compliance are crucial criteria for SSCM [3, 4, 15]. In this regard, concerns have been raised as to whether traditional information technology (IT) systems can facilitate and support the flow of sustainability details across multi-echelon SC (upstream and downstream) for the safe, transparent, and reliable authentication of product/process exchanges among SC partners [1, 14].

Emergent information and communication technologies (ICTs) have been touted as solutions to complex issues in SSCM, though the growing penetration of ICTs in SC activities has also led to increasing operational complexity for business across all sectors [16–19]. Specifically, emergent ICTs are undermining traditional workplace activities, forcing firms to change or modify SC strategies in light of digitization [20, 21]. Given that ICTs are becoming inextricably linked to SSCM [22], a new era of functional integration (e.g., enterprise resource planning system) involving blockchain tags that embed accountability and transparency in end-to-end SC transactions between members in a multi-tier SC is rapidly emerging [2, 23, 24]. A noteworthy exemplary is smart contracts, which are programs recorded on a blockchain that get activated when certain criteria are satisfied [9, 20]. Smart contracts are often used to automate the implementation of an agreement with efficiency and security, thereby instilling confidence among transacting parties with no intermediary participation or time wasted [12, 14].

Blockchain is a state-of-the-art technology emerging from the Fourth Industrial Revolution (IR4.0) [25–28] that holds immense potential in the digitization of SC [29, 30], with features such as data immutability, operational consistency, record tracking, and a consensus mechanism that creates a trusted business ecosystem built on cryptographic evidence with fewer or no intermediaries [1, 31]. Specifically, blockchain operates on a highly secure and live distributed ledger database that facilitates informational exchanges among SC partners to allow them to track the assembly of products from the moment they are procured until they reach the end user [28, 32, 33]. For example, the distribution of COVID-19 vaccines worldwide has been powered by blockchain, enabling manufacturers to proactively monitor its delivery and manage undesirable incidents (e.g., drug recall), and instilling a sense of confidence among consumers in the traceability of the vaccines that they receive [34, 35]. In this regard, blockchain empowers all members in the SC with real-time visibility into SC activities, thereby optimizing inventory management and improving response to SC issues [28, 36–38]. More importantly, incorporating cutting-edge ICTs such as blockchain in SSCM can address not only the barriers to accountability and traceability in SC [39], but also promote coordination and improve transparency as SC members around the world become more integrated through its use [32, 40, 41].

As policymakers and regulators increase pressure on focal firms to take sustainability actions, focal firms must direct each member in their SC to follow a common set of sustainability guidelines, which must be traceable, validated, and authenticated by SC members within focal firms’ network [42, 43]. Using cryptographic keys, blockchain is capable of recording all transactions within a SC network, protecting it with a hash pointer function in each block of transaction [43–46]. In other words, blockchain operates on secured frameworks necessary for sustainability management within the SC network to prevent data falsification by SC members or cyberattacks such as identity impersonation and sybil attacks [47–49]. The distributed and irreversible ledger of blockchains also renders transactions among SC partners and focal firm irremovable, thereby providing a complete record where every activity within the SC can be tracked for compliance in the long run [5, 50]. In this regard, blockchain provides visibility and transparency while protecting privacy of SC exchanges among SC partners and focal firms [51–53], thereby significantly contributing to SSCM [33, 54, 55].

To gain a deep and high-quality understanding of the application of blockchain for SSCM, this paper conducts a systematic literature review of articles published in high-quality journals to unpack the distinctive peculiarities characterizing high-quality research at the intersection of blockchain and SSCM. The focus on “high-quality” is important given the increasing importance of quality in scholarly research and the rise of predatory journals [43]. Though several reviews on blockchain and SC management avail, which were mostly concerned about the present state of blockchain adoption and future opportunities in SC [23, 48, 49, 56], and the technical advantages and challenges of blockchain implementation in SC [33, 39, 47, 57], few reviews sought to explore and link blockchain’s technological capability to SC sustainability, albeit scantly [39, 43, 56], with only a single review involved in an explicit investigation, albeit narrowly to the circular economy [33] (see Table 1). Noteworthily, no review, to date, has attempted to take stock of the extant literature relating to the application of blockchain for SSCM, which leaves understanding of the utility of blockchain for sustainable practices in SC incomplete. Similarly, no review has focused solely on articles published in high-quality journals, which leaves readers of reviews susceptible to low-quality insights of the field [43]. This is especially concerning in instances where multiple databases were used (see Table 1), which leaves greater room for errors and inefficiencies to manifest due to the large extent of duplication that emerges from such a review strategy [58, 59]. Finally, most reviews, to date, have overlooked the importance of delivering sector-specific insights, which have important implications for sector-specific adoption of blockchain for SSCM.

**Table 1:** Summary of existing reviews on blockchain and SC

Author(s)	Scope of review	Review type	Time period	Major findings
Gurtu and Johny, 2019 [23]	▪ Trend analysis of studies conducted on the potential of blockchain in SC management ▪ Analysis of 299 articles identified from EBSCO database	▪ Systematic literature review (structured narrative)	▪ 2015 to 2018	▪ Blockchain’s technical capabilities have enormous potential to reduce SC intermediaries and improve the efficiency of SC management ▪ Three major themes on blockchain and SC management research: (1) smart contracts, (2) SC finance, and (3) SC visibility and traceability
Wang, Han, and Beynon-Davies, 2019 [57]	▪ State of technological adoption of blockchain in SC ▪ Opportunities for blockchain in digitalized SC ▪ Challenges for successful diffusion of blockchain technologies in SC ▪ Analysis of 227 articles identified from nine integrated databases consisting of Emerald, IEEE Explore, ABI Inform Global, JSTOR, Web of Science, Scopus, Springer, ScienceDirect, and Taylor and Francis	▪ Systematic literature review (structured narrative)	▪ 2008 to 2017	▪ Blockchain innovation remains in an embryonic state but continues to gain traction in SC management, with trust serving as a primary motivator for their adoption ▪ Blockchain in SC management spans across (1) SC finance, (2) SC intermediation structure, (3) SC member relationship management, (4) SC sustainability, and (5) negative consequences of blockchain implementation ▪ Blockchain implementation raises (1) institutional, (2) technological, and (3) operational challenges
Dutta et al., 2020 [39]	▪ Current adoption status and implementation challenges and opportunities for blockchain technologies in the field of SC management across various business sectors ▪ Societal impacts of blockchain technologies Analysis of 178 articles identified from Scopus database	▪ Systematic literature review (structured narrative)	▪ 2017 to 2019	▪ Blockchain in SC management spans across (1) blockchain adoption and implementation in SC, (2) SC reengineering, (3) SC resilience, (4) SC coordination, (5) security enhancement, (6) business process management, (7) SC sustainability, and (8) sector specific peculiarities ▪ Challenges to implement blockchain for SC management include (1) organizational and (2) technical challenges ▪ Opportunities for societal impacts of blockchain implementation in SC include humanitarian SC, cryptocurrency for recycling activities, smart cities, social sustainability, and activities aimed at achieving circular economy goals
Müßigmann, Gracht, and Hartman, 2020 [49]	▪ Blockchain’s role and application in logistics and SC management ▪ Analysis of 613 articles identified from 10 different databases consisting of Scopus, Google Scholar, Web of Science, Springer, IEEE Xplore, ScienceDirect, SSRN, Taylor & Francis, EBSCO, and Emerald Insight	▪ Systematic literature review (bibliometric)	▪ 2016 to 2019	▪ Five distinct areas of research on blockchain for SC, namely (1) concept development about the challenges, opportunities, and barriers to blockchain technology adoption, (2) evaluating and conceptualizing frameworks for impact analysis of blockchain in industry-specific cases, (3) blockchain-based digitized SC, (4) technical design of blockchain applications for real-world applications, and (5) framing blockchain and other interdisciplinary technologies for SC management
Pournader et al., 2020 [48]	▪ Applications of blockchain technologies in SC, logistics, and transport management ▪ Analysis of 48 articles identified from Scopus and ISI Web of Knowledge databases	▪ Systematic literature review (bibliometric)	▪ 2016 to 2018	▪ Blockchain in SC, logistics, and transport management span across the 4Ts of blockchains, namely technology, trust, trade, and traceability/transparency
Wamba et al., 2020 [47]	▪ Advantages and drawbacks of bitcoin, blockchain, and fintech for SC management ▪ Analysis of 141 articles identified from five different databases consisting of Academic Search Complete, ABI/INFORM Complete, Emerald Journals, ScienceDirect, and JSTOR	▪ Systematic literature review (structured narrative)	▪ 2007 to 2017	▪ Bitcoin, blockchain, and fintech are constantly emerging and evolving for SC management across different industries ▪ Implementation benefits include (1) low transaction costs and (2) trustworthiness of the peer-to-peer transaction system ▪ Implementation barriers include (1) lack of legislative regulation for fintech and (2) absence of clear legal status for bitcoin
Wamba and Queiroz, 2020 [56]	▪ Assess the current status of research in blockchain for operations and SC management ▪ Future research directions for blockchain technologies and how they interact with SC and operations management activities across sectors ▪ Analysis of articles (exact number not specified) identified from Web of Science database	▪ Systematic literature review (bibliometric)	▪ 2013 to 2020	▪ Guest editorial inviting prospective researchers to investigate the technological role of blockchain across various sectors such as food, e-commerce, and healthcare ▪ Future studies should concentrate on evaluating the effect of blockchain technologies on the environment and sustainable business activities
Lim et al., 2021 [43]	▪ Future prospects for tertiary industries’ blockchain-based SC on sustainable themes ▪ Analysis of 106 articles identified from Web of Science database	▪ Systematic literature review (structured narrative)	▪ 2017 to 2020	▪ Blockchain technologies for SC management activities spans across four categories, namely (1) neglected concepts in SC such as environmental issues, social sustainability, and economic dimensions, (2) usage of new research methods, such as conceptual, empirical, modeling, and technical approaches to gain a better understanding of blockchain’s applicability in SC, (3) academic theory and industrial practice that reflect the development of new theories for real-world application and analysis using a case study approach, respectively, and (4) its implementation across different industrial sectors
Moosavi et al., 2021 [30]	▪ Application of blockchain technologies for different areas of SC ▪ Analysis of 286 research articles identified from Scopus and Web of Science databases	▪ Systematic literature review (bibliometric)	▪ 2010 to 2019	▪ New research aspirations should focus on the creation of a blockchain deployment framework that makes use of internet of things technologies and machine learning algorithms ▪ Future studies should concentrate on assessing the sustainability-, resiliency-, reliability- and flexibility aspects of blockchain technologies
Tandon et al., 2021 [29]	▪ Applications of bitcoin and blockchain technologies for business management ▪ Analysis of 586 articles identified from Scopus database	▪ Systematic literature review (bibliometric)	▪ 2015 to 2019	▪ Applications of blockchain technologies for business management spans across four areas, namely (1) strategic and regulatory issues affecting bitcoin and blockchain implementation, (2) benefits and drawbacks of integrating blockchain into business frameworks, (3) using blockchain technologies across diverse organizational disciplines to build a productive manufacturing ecosystem, and (4) inefficiencies of bitcoin ▪ Future studies are encouraged in five areas, namely (1) extending the sectoral scope of blockchain’s application, (2) blockchain applications for developing economies, (3) effect of the business environment on inter- and intra-institutional blockchain deployment, (4) consumer’s perspective of blockchain application and awareness of technology, and (5) development of a research framework for empirical validation based on management theories such as UTAUT, diffusion of innovation, and behavioral resistance theory
Upadhyay et al., 2021 [33]	▪ Present retrospective and prospective contributions of blockchain technologies to the circular economy in the domain of business management through the viewpoint of sustainability and social responsibility ▪ Issues and problems in implementing blockchain technologies for the circular economy ▪ Analysis of research articles (exact number not specified) identified from Web of Science, Cross Ref, EBSCO Business Source Premier, and Science Direct databases	▪ Systematic literature review (structured narrative)	▪ 2015 to 2020	▪ Blockchain systems, with their peer-to-peer verification and participative properties in SC management, serve to drive the circular economy ▪ Blockchain, in particular, will contribute to the circular economy by lowering transaction costs, increasing productivity, securing connectivity along the SC, ensuring human rights compliance, and reducing environmental impact ▪ Challenges of incorporating blockchain in terms of achieving circular economy objectives include the initial cost of technical adoption, illegal activities, and lack of regulatory governance ▪ Future research should explore the following questions: (1) how blockchain technology may apply within the circular economy paradigm of social responsibility, (2) how country-specific regulations affect blockchain development and deployment to realize circular economy goals, and (3) how will developing countries with infrastructural constraints utilize blockchain technologies to build circular economies?

To achieve its aim and address the extant gaps of past reviews, this paper conducts a systematic literature review that delivers a state-of-the-art overview of high-quality research on blockchain for SSCM inclusive of sector-specific insights using a bibliometric analysis, which is an objective review technique, of articles published in journals ranked “A*”, “A”, and “B” by the Australian Business Deans Council, which represents the journal ranks reflective of “high quality”. The articles and their bibliometric details will also be retrieved using Scopus, which is one of the most comprehensive scientific databases, for the bibliometric analysis. The use of a single scientific database was recommended to reduce unintentional mistakes caused by multiple databases, such as double counting from duplicate entries. In line with past systematic literature reviews relying on a bibliometric analysis [60–64], this study seeks to shed light on the answers to the following research questions (RQs):

**RQ1.** What are the performance trends of high-quality research publications, citations, and constituents (authors, institutions, countries) on blockchain for SSCM?

**RQ2.** What are the major themes of high-quality research on blockchain for SSCM?

**RQ3.** What are the sector-specific insights of high-quality research on blockchain for SSCM?

**RQ4.** What are the potential uses of blockchain for SSCM across various sectors indicated by high-quality research in the field?

**RQ5.** What are the research gaps and questions on blockchain for SSCM that warrant future research?

The remainder of this paper is organized as follows. The paper begins with an overview of the fundamentals of blockchain in relation to SC and SSCM. This is followed by the methodological aspects of its systematic literature review, and the ensuing findings from its review. The paper concludes with suggestions to fertilize the field with high-quality research on blockchain for SSCM.

## Theoretical foundation

### Blockchain for SC

**Fig. 1:**
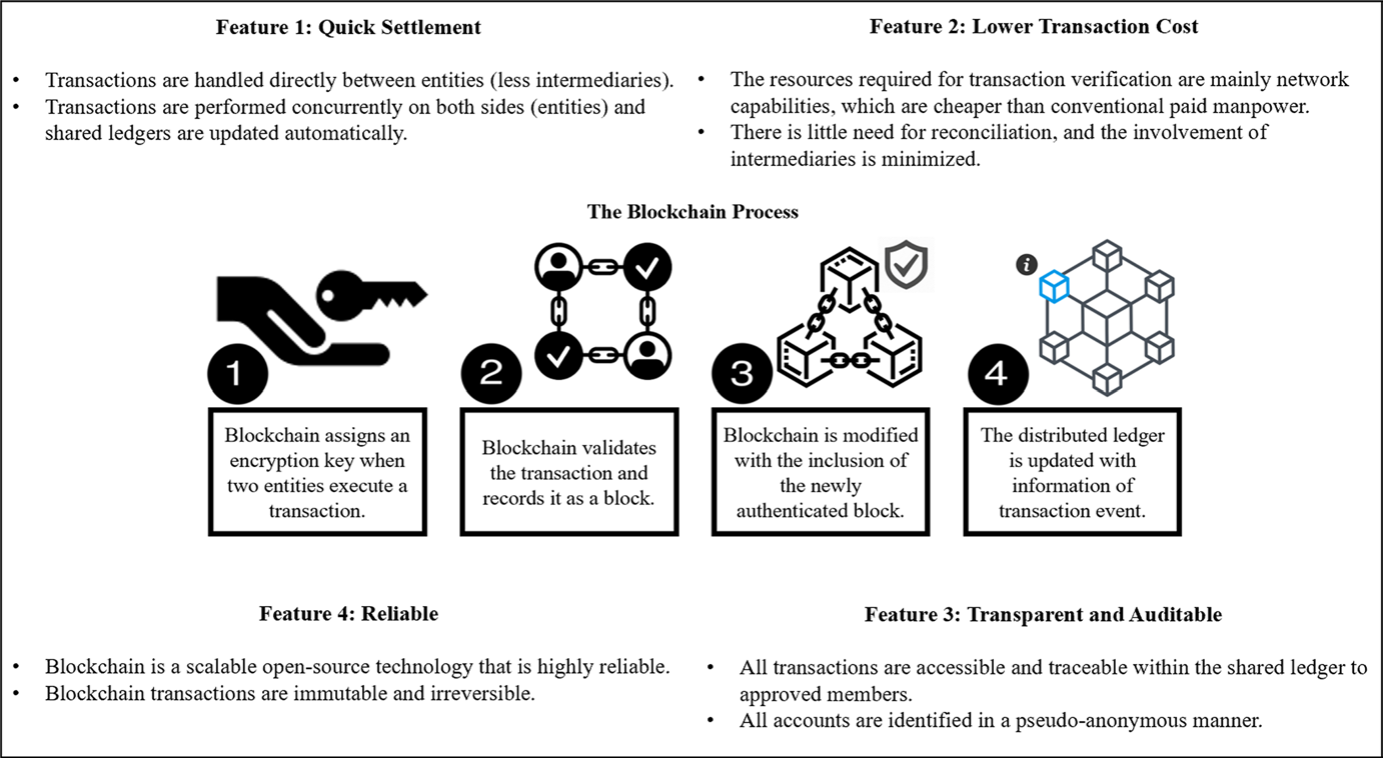
The key features and process of blockchain transaction

Blockchain attracted global attention when its spinoff, bitcoin, disrupted the financial market [65, 66], with other spinoffs in new areas emerging rapidly, such as healthcare [67], oil and gas [68], and telecommunications [69]. This IR4.0 technology serves as an essential enabler of SC in the circular economy[16, 33, 70, 71]. Indeed, SC has become increasingly complicated as a result of globalization, involving participants worldwide and requiring a great deal of interorganizational coordination [50, 72]. This raises the cost of SC management, especially for focal firms engaged in international business transactions [5, 7, 17, 46]. Nonetheless, blockchain is a promising solution for lowering transaction costs in SC management [33], with the process of a blockchain transaction between two business entities illustrated in Fig. 1.

In essence, blockchain is a cryptographic digital ledger or an electronically protected archive of documents, interactions, or performed activities that are exchanged by participating members [31, 73, 74]. Operational activities based on blockchain facilitates the authenticated exchange of information between each entity in a SC without the need for a trustworthy centralized authority to act as an intermediary [75–77]. In this regard, blockchain offers a quick settlement that lowers transaction cost and increases transparency across a SC by confirming and recording data in real time [14, 24, 78, 79]. That is to say, blockchain is a global platform that business entities can rely upon to record SC transactions or other digital interactions in a way that is encrypted, accessible, completely immune to outages, auditable, and reliable [23, 39].

Global SC networks are currently governed by information systems, with transaction data maintained in databases, posing significant risks that blockchain could mitigate. When comparing blockchain and database (Table 2), the first noticeable difference is centralization. Database functions in a centralized manner, whereas blockchain functions in a decentralized way [78], and thus, blockchain has the potential to change the existing state of information systems employed in SCs [80]. Though decentralization necessitates substantial changes to existing information systems used for SC management by different sectors, it can empower all members in a SC to operate independently and eliminates the need for centralized control [81, 82]. For example, a blockchain-enabled smart contract is a self-executing contract in which the conditions of the buyer–seller agreement are directly encoded into lines of code [23, 40]. The code and the agreements contained within it are dispersed and decentralized over a blockchain network [46].

**Table 2:** Comparison of blockchain versus database

Attribute	Information technology	
	Blockchain	Database
Authority	Decentralized distributed ledger technology that works on a peer-to-peer approach	Centralized ledger in which data is stored in a systematic manner and is controlled by an administrator
Architecture	Uses a distributed ledger network architecture	Uses a client–server architecture
Data handling	Data management using an authentication approach supported by timestamp-enabled read and write functions	Data management with no authentication mechanism that supports create, read, update, and delete functions
Integrity	Supports data integrity where any malicious act is recorded	Malicious actor can alter data in the database
Transparency	Offers transparency among participants	Not transparent as administrators decide which participants have access to data
Functionality	An emerging technology that is complex to deploy and maintain independently, though its functionality can be democratized to and used by all participants	A mature and well-established technology that is easy to deploy and maintain independently, but its functionality is confined to independent participants
Performance	Operates on verification and consensus mechanism at a larger scale	Operates on internal mechanism at a small scale

Another advantage of blockchain over database is that it allows immutability, which implies that data, once recorded, cannot be wiped or updated [55, 82]. In this regard, data in blockchain becomes trustworthy as it can autonomously detect and rectify itself based on programmed business logic and consensus [39]. Noteworthily, it is practically never the case that two organizations collaborate on a single database containing a single set of entries, since database, in most cases, is managed and updated by the database administrator of the focal company [83]. Due to the fact that only one out of two organizations pays the database administrator, there is vested interest to attain the success of the paying organization, but not necessarily that of the other organization. In other words, if the paying party makes a move that favors their organization, the other party or organization will never know. Similarly, and perhaps more dangerously, if a business rival chooses to pay off the database administrator, then that rival may make modifications to the database without either transacting organizations knowing [83].

When blockchain technology is used in the data transaction process, it also eliminates the single point of failure, which means that if one of participant makes a mistake, the other participants can quickly restore it, since each participant keeps their own digitally encrypted ledger [66, 74]. After the data in the blockchain-based ledger has been rectified, the unchangeable record of alteration will reveal which participant has made the change [84–86]. With the data process safeguarded, an organization can rely not only on the information exchanged between collaborating organizations but also on the information supplied by competitors in the SC ecosystem. This exemplifies the usage of smart contract technologies and the power of its encryption [46, 73], resulting in lower costs and accuracy since there are no middlemen or cost fees throughout the execution process, as well as no human interference during the execution process [39, 67, 87].

### Blockchain for SSCM

**Fig. 2:**
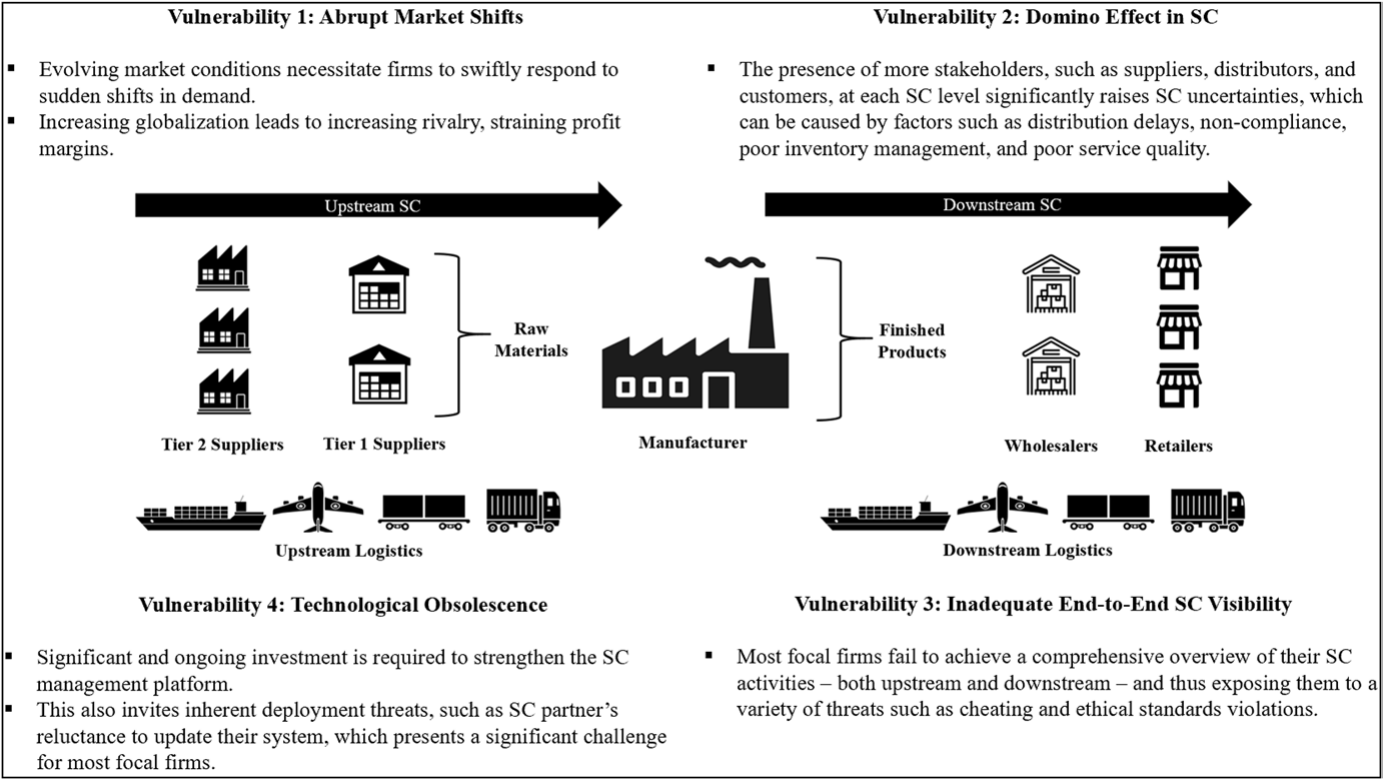
The key vulnerabilities in multi-layer SC

The complexity of new-age SC has escalated [88], and the impact of its management on business competitiveness is recognized as an aspect that warrants further investigation [20, 32, 76]. To illustrate, the SC for a typical focal firm (manufacturer) engaged in multi-layered relationships of upstream and downstream physical flows [1, 4, 12], alluding to the notion of a multi-echelon SC [2, 3, 18], is presented in Fig. 2. Specifically, firms today are under immense pressure to engage in sustainable practices across the SC as a result of recent developments such as globalization, market shifts, demand uncertainty, and economic challenges, and thus, relying solely on internal efficiencies of SC is now inadequate to gain a competitive edge [14, 78, 89]. Moreover, the restructuring practices engaged by any one firm in transforming traditional SC management into SSCM can place an equivalent pressure on its SC partners to improve their own practices to meet sustainability requirements of the reinvigorated SC [5, 42]. Therefore, the operational complexities and vulnerabilities in the contemporary, multi-echelon SC are affected by a variety of internal and external factors [2–5, 7], as illustrated in Fig. 2.

Many focal firms in multi-echelon SC networks that are engaged in SSCM—either proactively or reactively as a result of enforcement by regulatory authorities or pressure by stakeholders [5, 12, 48]—face the challenge of monitoring and tracing of SC activities [3, 7]. To overcome this challenge, it is important that every SC member provides authentic and timely information of their SC activities [50], which is nonetheless dependent on the system capacity of each SC member to accurately gather and record data and produce reliable reports to the systems of other SC members in a secured manner [3, 5, 7]. Most often, the effectiveness of information flow in an end-to-end SC for a focal firm is limited by the SC’s weakest member, or the “most immoral” link. Because interorganizational operability standards in a multi-echelon SC are constrained by external factors, there is often little flexibility in the provision of information pertaining to sustainability compliance among SC members [14, 45]. Due to increasing complexity of interactions among SC members, it is equally challenging for focal firms to monitor and trace details of product movement without distortion [18, 46, 70]. Most SC members struggle to access information from other SC members due to the lack of secured information sharing infrastructure, which is exacerbated by the fact that information is fragmented across several stakeholders [39, 73, 90]. The presence of diverse stakeholders in a multi-echelon SC is also correlated with the possibility of fraudulent cases caused by unethical members, raising business risks such as reputational damage and financial losses for participating firms [5, 7]. Identifying code of ethics violations in SC is now one of the most pressing issues that firms today must address [5, 8, 75]. This implies that the effectiveness of SSCM is determined by the level of trust established and information sharing among stakeholders [45].

Blockchain offers an innovative way out to address the inherent issues of trust and information sharing of sustainability practices among SC members through its inherent technological qualities, supported by internet-of-things (IoT) technologies [9, 50]. Noteworthily, the unchangeable and irreversible characteristics of blockchain can ensure traceability and reduce the risks involved by efficiently exchanging information among stakeholders involved in the multi-echelon SSCM [39, 41, 47]. Furthermore, the transparent and auditable functionality of blockchain enabled by suitable IoT technology can provide requisite access to information in the future for focal firms to address accountability issues in event of non-compliance by an SC member [57, 90, 91]. In addition, blockchain operates on self-enforcing consensus among SC members to render a transaction to be authentic, and thus, preventing false or potentially illegitimate transactions from being recorded in the digitally distributed ledger [12, 53]. Moreover, blockchain is a scalable application built for peer-to-peer networks that allows for quick financial settlements between SC members while eliminating the need for trusted intermediaries [23, 47, 63, 92, 93]. This smart contract functionality in blockchain is critical in lowering economic and reputational harm [54, 73, 74].

Taken collectively, implementing blockchain systems can make it easier for focal firms to collect information (e.g., certification, date, location, price, quality) from their SC partners and thus improve SSCM [11, 94]. In other words, the availability of information through a blockchain-based infrastructure for a multi-layered SC, as seen in Fig. 2, can improve the traceability of products in the SC [9, 44], lower losses from counterfeit and grey markets [14, 49, 54], boost visibility and compliance over outsourced contract manufacturing [39, 57], and strengthen the focal firm’s role as a leader in responsible manufacturing [2].

## Methodology

This paper conducts a systematic literature review using a bibliometric methodology, whereby bibliometric information of articles is collected as data and used as input in a bibliometric analysis [58, 95, 96]. Specifically, the bibliometric methodology encapsulates the use of a set of quantitative techniques on bibliometric information to assess the performance of a corpus of articles that represent a field of study, unpack the major themes that underpin the intellectual structure of that field, and inform future research about the extant gaps and potentially fruitful avenues to drive the field forward [29, 30, 49, 58, 97, 98, 99]. The data collection and analysis methods and procedures for the bibliometric methodology adopted and implemented herein are explained in the next sections.

### Data collection method and procedure

The first task in conducting a systematic literature review is to identify relevant keywords to be used as query input into the scientometrics database. For this purpose, this review relied on Google Scholar to identify suitable synonymous keywords related to three groups of focal keywords that characterize the topic of the current investigation: “blockchain”, “sustainability”, and “supply chain”. A separate search query was conducted for each keyword on Google Scholar, with the search period limited to the past five years for the purpose of recency and relevance, returning several pages of meta-results with 10 articles listed on each page. The articles from the top 10 pages—i.e., the top 100 articles—were used to extract the synonymous keywords that are related to the focal keyword, which were subsequently stored in an Excel file. On the basis of frequency count, the synonymous keywords relating to the three focal keywords that form the foundation of the present inquiry were chosen to be used in the search query on Scopus. The list of synonymous keywords were also discussed and agreed upon by the research team.

This systematic literature review acquired its data on November 15, 2021 for the period up to October 2021 from Scopus, which is the world’s largest, high-quality repository of bibliometric information for scientific articles [29, 39, 100, 101]. Specifically, this review employed a three-stage content filtration strategy, which includes (1) specifying relevant search terms using Boolean operators [30, 47, 49], (2) stating the criteria for document screening [39, 43, 48], and (3) compiling a corpus of articles based on source quality [23, 29] (see Table 3).

**Table 3:** Schema of data collection

Content filtration stage (CFS)	Action item		Results (Articles)
CFS 1	Search string: Blockchain-related keywords	“blockchain” OR “cryptographic ledger” OR “digital ledger” OR “distributed ledger” OR “public transaction ledger”	1309
		AND	
	Sustainability-related keywords	“sustainable” OR “sustainability” OR “green” OR “environment*” OR “social*” OR “economic*” OR “circular economy”	
		AND	
	Supply chain-related keywords	“supply chain” OR “supply chain management” OR “logistics” OR “transport*” OR “mobility”	
CFS 2	Limit to: ▪ Document type: “Article” ▪ Source type: “Journal”		556
CFS 3	Article filtration by high-quality journals (ABDC “A*”, “A”, and “B”):		146
	1. *International Journal of Production Research* (A) 2. *Journal of Cleaner Production* (A) 3.*Computers and Industrial Engineering* (A) 4. *Transportation Research Part E Logistics and Transportation Review* (A*) 5. *IEEE Transactions on Vehicular Technology* (A) 6. *IEEE Transactions on Intelligent Transportation Systems* (A) 7. *Technological Forecasting and Social Change* (A) 8. *IEEE Transactions on Engineering Management* (A) 9. *International Journal of Production Economics* (A) 10. *International Journal of Information Management* (A) 11. *Journal of Enterprise Information Management* (A) 12. *Industrial Management and Data Systems* (A) 13. *Supply Chain Management: An International Journal* (A) 14. *Annals of Operations Research* (A) 15.*Business Strategy and the Environment* (A) 16. *Computers and Security* (A) 17.*British Food Journal* (B) 18.*International Journal of Logistics Research and Applications* (B)	19. *International Journal of Productivity and Performance Management* (B) 20. *Journal of Business Research* (A) 21. *Journal of Organizational Change Management* (B) 22. *Journal of Theoretical and Applied Electronic Commerce Research* (B) 23. *Marine Policy* (A) 24. *Production Planning and Control* (A) 25. *Applied Economic Perspectives and Policy* (B) 26. *Applied Energy* (A) 27. *Automation in Construction* (A*) 28. *California Management Review* (A) 29. *Cogent Economics and Finance* (B) 30. *Computers and Operations Research* (A) 31. *Current Issues in Auditing* (B) 32. *Decisions in Economics and Finance* (B) 33. *Disasters* (A) 34. *Economics of Innovation and New Technology* (B) 35. *Engineering Construction and Architectural Management* (B) 36. *Information Processing and Management* (B) 37. *Information Systems and E-business Management* (B) 38. *Information Systems Frontiers* (A) 39. *Information Systems Management* (B) 40. *Information Technology and People* (A) 41. *Information Technology for Development* (B)	

Search database = Scopus. Search date = November 15, 2021. Search period = up to October 31, 2021

In the first stage of content filtration (CFS1), three groups of keywords relating to “blockchain”, “sustainability”, and “supply chain” were identified from previous studies [5, 33, 39, 48, 49] and used in a search within “article title, abstract, and keywords” for publications related to blockchain for SSCM on Scopus. This resulted in a total of 1,309 documents.

In the second stage of content filtration (CFS2), the documents were filtered based on document and source type, whereby only documents that are classified as an “article” and published in sources identified as a “journal” were included. Other document types such as “book”, “book chapter”, “conference paper”, “conference review”, “editorial”, “note”, “review”, and “short survey” and other source types such as “book”, “book series”, “conference proceeding”, and “trade journal” were excluded due the lack of rigorous peer review scrutiny and quality thresholds [58, 59]. This resulted in 556 documents, which will be referred to as articles from this point onwards.

In the third and final stage of content filtration (CFS3), the articles were filtered based on source quality, wherein articles published in journals ranked “A*”, “A”, or “B” by the Australian Business Deans Council (ABDC) were included for review. The ABDC describes journals ranked “A*”, “A”, and “B” as “high-quality” journals that have been recommended by the council’s expert panel [102], and thus, represent the best journals in the field [23]. This laser focus on high-quality journals also enables the review to avoid the pitfall of including any dubious and predatory journals that could avail [103–105]. As a result, a total of 146 articles were identified and included in the final corpus for review.

### Data analysis method and procedure

**Fig. 3:**
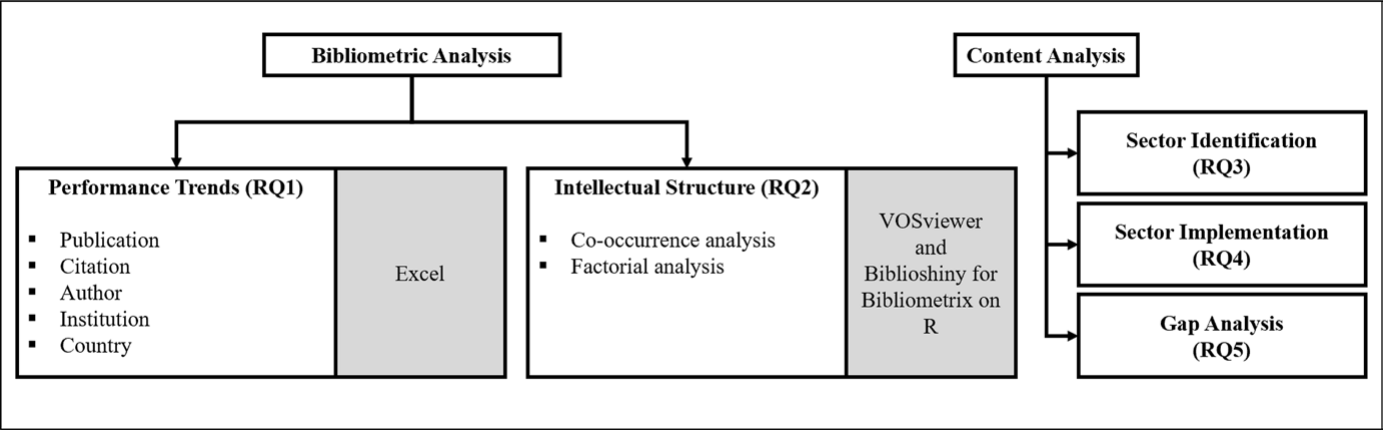
Schema of data analysis

This systematic literature review conducted a bibliometric analysis on the corpus of 146 articles that were retained (see Fig. 3). In line with the two-stage analytical technique (i.e., performance analysis and science mapping) recommended by Donthu et al. [58] for bibliometric research, this review performed a performance analysis to delineate the performance trends of publications, citations, and constituents (authors, institutions, countries), and a co-occurrence analysis and a factorial analysis as part of science mapping to unpack the major themes that underpin the intellectual structure of high-quality research on blockchain for SSCM. The performance analysis was carried out using Excel to answer RQ1, whereas the co-occurrence analysis was conducted using VOSviewer [96, 106] and the factorial analysis was performed using Biblioshiny for Bibliometrix on R [107] to answer RQ2. Following the discovery of major themes using these quantitative techniques, a qualitative content analysis was conducted to source for sector-specific insights to answer RQ3, RQ4, and RQ5.

## Findings

### Performance analysis

#### Publication and citation trend of blockchain for SSCM research

The publication and citation trend of the corpus of blockchain for SSCM research is presented in Table 4. In total, the corpus consists of 146 articles published in 41 journals ranked “A*”, “A”, and “B” by the ABDC in 2019.

**Table 4:** Publication and Citation Trend of Blockchain for SSCM Research

Journal title	Category (ABDC)	2018	2019	2020	2021 (Oct.)	TP	TC	C/P	h
*International Journal of Production Research*	A		1	8	8	17	741	43.58	10
*Journal of Cleaner Production*	A			4	9	13	132	10.15	7
*Computers and Industrial Engineering*	A		4	1	6	11	204	18.54	7
*Transportation Research Part E: Logistics and Transportation Review*	A*		2	3	5	10	249	24.90	7
*IEEE Transactions on Vehicular Technology*	A			4	5	9	111	12.33	4
*IEEE Transactions on Intelligent Transportation Systems*	A	1			6	7	237	33.85	3
*Technological Forecasting and Social Change*	A			3	4	7	60	8.57	4
*IEEE Transactions on Engineering Management*	A			2	4	6	8	1.33	2
*International Journal of Production Economics*	A			3	3	6	177	29.50	4
*International Journal of Information Management*	A*			4	1	5	282	56.40	4
*Journal of Enterprise Information Management*	A		1	3	1	5	65	13.00	4
*Industrial Management and Data Systems*	A			1	3	4	22	5.50	3
*Supply Chain Management: An International Journal*	A	1	1	1	1	4	285	71.25	3
*Annals of Operations Research*	A				3	3	2	0.67	1
*Business Strategy and The Environment*	A				3	3	7	2.33	1
*Computers and Security*	A		1	1	1	3	149	49.67	2
*British Food Journal*	B				2	2	0	0.00	0
*International Journal of Logistics Research and Applications*	B				2	2	23	11.5	1
*International Journal of Productivity and Performance Management*	B				2	2	0	0.00	0
*Journal of Business Research*	A				2	2	16	8.00	1
*Journal of Organizational Change Management*	B				2	2	3	1.50	1
*Journal of Theoretical and Applied Electronic Commerce Research*	B				2	2	2	0.00	0
*Marine Policy*	A			2		2	65	32.50	2
*Production Planning and Control*	A			2		2	19	9.5	2
*Applied Economic Perspectives and Policy*	B				1	1	3	3.00	1
*Applied Energy*	A				1	1	16	16.00	1
*Automation in Construction*	A*		1			1	97	97.00	1
*California Management Review*	A				1	1	0	0.00	0
*Cogent Economics and Finance*	B			1		1	23	23.00	1
*Computers and Operations Research*	A			1		1	74	74.00	1
*Current Issues in Auditing*	B				1	1	0	0.00	0
*Decisions in Economics and Finance*	B				1	1	0	0.00	0
*Disasters*	A			1		1	6	6.00	1
*Economics of Innovation and New Technology*	B				1	1	2	2.00	1
*Engineering Construction and Architectural Management*	A				1	1	27	27.00	1
*Information Processing and Management*	B				1	1	39	39.00	1
*Information Systems and E-Business Management*	B				1	1	0	0.00	0
*Information Systems Frontiers*	A				1	1	1	1.00	1
*Information Systems Management*	B			1		1	6	6.00	1
*Information Technology and People*	A				1	1	4	4.00	1
*Information Technology for Development*	B				1	1	0	0.00	0
Total	3 A*, 24 A, 14 B	2	11	46	87	146	3,157	21.62	

*Note* *TP* total publications, *TC* total citations, *C/P* citations per publication, *Oct.* October. *h* *h*-index

The table indicates that blockchain for SSCM research is relatively new, with the field’s first two articles in high-quality journals appearing only in 2018. Nonetheless, the field’s high-quality productivity is exponential, with articles in “A*”, “A”, and “B” journals increasing three-fold in 2019 (*n* = 11), and six-fold in 2020 (*n* = 46). In the first 10 ½ months of 2021, 87 articles were published in “A*”, “A”, and “B” journals, indicating that the proliferation of high-quality blockchain for SSCM research is projected to be at least 40-fold as compared to articles in 2018 when the year 2021 comes to a close. Noteworthily, the growth of blockchain for SSCM research from 2020 to 2021 (± 90%) appears to be close to double of the global blockchains services market that is expected to grow from $1.06 billion to $1.62 billion within the same period (± 50%) [108], which indicates that research interest is greater than the actual growth of its application in practice.

The impact and influence of blockchain for SSCM research in high-quality journals are also noteworthy, as indicated by the average of 21.62 citations per publication and the total of 3,157 citations garnered for the 146 articles in the area that have been published in “A*”, “A”, and “B” journals within the last four years.

The most productive high-quality journal on blockchain for SSCM research is *International Journal of Production Research* with 17 articles, followed by *Journal of Cleaner Production* and *Computers and Industrial Engineering* with thirteen and eleven articles, respectively. *International Journal of Production Research* is also the most influential high-quality journal in the field, with a total of 741 citations for 17 articles. The journal also has the highest *h*-index of 10, which indicates that 10 of its articles in the field have received at least 10 citations from other articles indexed in Scopus within the last four years. Nonetheless, *Supply Chain Management: An International Journal* is the most impactful high-quality journal in the field, with an average of 71.25 citations from other articles indexed in Scopus for the four articles that the journal has published over the last four years.

#### Top contributing authors of blockchain for SSCM research

The top contributing authors of blockchain for SSCM research in high-quality journals are listed in Table 5. In total, 16 out of 460 authors have published at least three articles on blockchain for SSCM research in high-quality journals. In terms of research productivity, J. Sarkis is the most prolific author on the list with eight articles, followed by A. Gunasekaran with four articles. In terms of research impact and influence, J. Sarkis leads the list in terms of cumulative citations, with 667 citations from eight papers, followed by M. Kouhizadeh, who has 620 citations from three articles. M. Kouhizadeh also has the greatest average number of citations, with 206.67 citations per article. In terms of author dominance reflected by the author’s dominance factor (ADF) ratio, which considers the number of times an author acts as first author in a multi-authored article and thus indicates the author’s dominant position in publishing articles [100], both T. M. Choi and B. Niu emerge as authors with the highest ADF of 1.00. Noteworthily, the number of unique top contributing authors with at least three articles between 2019 and 2021 have increased over the years, from four unique top contributing authors in 2019 to 13 and 15 unique top contributing authors in 2020 and 2021, respectively, thereby indicating that the growth of blockchain for SSCM research coincides with the growth of unique top contributing authors.

**Table 5:** Top contributing authors on blockchain for SSCM research

Author	Measure							
	TP	FA	ADF	TC	C/P	TP/Y and JT		
						2019	2020	2021 (Oct.)
J. Sarkis	8	0	0	667	83.37	1	1	6
						*IJPR*	*IJPR*	*IJLRA, IJPE, IJPR, IMDS, ITEM*
A. Gunasekaran	4	0	0	207	51.75		2	2
							*COR*, *IJIM*	*CIE, TFSC*
C. Bai	3	2	0.67	90	30.00		1	2
							*IJPR*	*IJPE, ITEM*
T. M. Choi	3	3	1.00	144	48.00	1	2	
						*TRE*	*IJPE, TRE*	
G. Epiphaniou	3	1	0.33	5	1.67		1	2
							*ITEM*	*CS, ITEM*
G.Q. Huang	3	0	0	49	16.33	1	1	1
						*CIE*	*IJPR*	*CIE*
S. S. Kamble	3	2	0.67	203	67.67		2	1
							*COR*, *IJIM*	*TFSC*
M. Kouhizadeh	3	1	0.33	620	206.67	1		2
						*IJPR*		*IJPE, IJPR*
N. Kumar	3	0	0	119	39.67	1	1	1
						*CS*	*ITVT*	ITVT
V. Kumar	3	0	0	106	35.33		1	2
							*COR*	*JCP*, *TFSC*
Z. Li	3	1	0.33	43	14.33		2	1
							*CIE, IJPR*	*IJPR*
M. K. Lim	3	1	0.33	19	6.33		1	2
							*IMDS*	*CIE, IMDS*
S. Nandi	3	2	0.67	38	12.67		1	2
							*SCMJ*	*IJLRA, IMDS*
B. Niu	3	3	1.00	13	4.33			3
								*JCP, TRE*
R. Sharma	3	2	0.67	198	66.00		2	1
							*COR, IJIM*	*JEIM*
Z. Yu	3	0	0	28	9.33			3
								*ANOR, BSE, IJLRA*

**Note(s):** Minimum of three articles for author inclusion. TP = Total Publications. FA = Number of First-authored Publications. ADF = Author’s Dominance Factor = Number of Publications as First Author ÷ Number of Multi-authored Publications. TC = Total Citations. C/P = Citations per Publication. TP/Y = Total Publications per Year. JT = Journal Title. Oct. = October. *ANOR* = *Annals of Operations Research. BSE* = *Business Strategy and the Environment. CIE* = *Computers and Industrial Engineering. COR* = *Computers and Operations Research. CS* = *Computers and Security. IJIM* = *International Journal of Information Management. IJLRA* = *International Journal of Logistics Research and Applications. IJPE* = *International Journal of Production Economics. IJPR* = *International Journal of Production Research. IMDS* = *Industrial Management and Data Systems. ITEM* = *IEEE Transactions on Engineering Management. ITVT* = *IEEE Transactions on Vehicular Technology. JEIM* = *Journal of Enterprise Information Management. TFSC* = *Technological Forecasting and Social Change. TRE* = *Transportation Research Part E: Logistics and Transportation Review. SCMJ* = *Supply Chain Management: An International Journal*

#### Top contributing institutions of blockchain for SSCM research

The top contributing institutions of blockchain for SSCM research in high-quality journals are listed in Table 6. In total, 10 out of 280 institutions have published at least four articles on blockchain for SSCM research in high-quality journals. Worcester Polytechnic Institute (United States) is the most productive institution on the list in terms of research output, with eight articles published in five journal titles. Hanken School of Economics (Finland) ranks second on the list, with six articles published in five journal titles. Joint-third on the list are University of Electronic Science and Technology of China (China), Hong Kong Polytechnic University (Hong Kong), and University of Hong Kong (Hong Kong), all of which have five articles to their credit. California State University (United States), Chang’an University (China), Coventry University (United Kingdom), Manchester Metropolitan University (United Kingdom), and Shenzhen University (China) are all on the list with four articles. In terms of research impact and influence, Worcester Polytechnic Institute has attracted the most citations (TC = 760) from eight articles, followed by California State University and University of Electronic Science and Technology of China with a total of 217 and 151 citations from four and five articles, respectively.

**Table 6:** Top contributing institutions on blockchain for SSCM research

Institution	Measure	2019	2020	2021 (Oct.)	TC
Worcester Polytechnic Institute, United States	TP	1	1	6	8
	JT	*IJPR*	*IJPR*	*IJLRA, IJPE, IJPR, IMDS, ITEM*	5
	TC	579	87	94	760
Hanken School of Economics, Finland	TP		1	5	6
	JT		IJPR	*IJLRA, IJPE, IJPR, IMDS, ITEM*	5
	TC		87	17	104
University of Electronic Science and Technology of China, China	TP		2	3	5
	JT		*IJPR, ITVT*	*CIE, IJPE, ITEM*	5
	TC		144	7	151
Hong Kong Polytechnic University, Hong Kong	TP	1	1	3	5
	JT	CIE	*IJPR*	*CIE, IJPR, JCP*	3
	TC	28	17	26	71
University of Hong Kong, Hong Kong	TP	1	1	3	5
	JT	CIE	*IJPR*	*CIE, IJPR, JCP*	3
	TC	28	17	26	71
California State University (Bakersfield), United States	TP		2	2	4
	JT		*COR, IJIM*	*CIE, TFSC*	4
	TC		206	11	217
Chang’an University, China	TP		1	3	4
	JT		*IMDS*	*ANOR, BSE, IJLRA*	4
	TC		4	30	34
Coventry University, United Kingdom	TP		1	3	4
	JT		*IMDS*	*ANOR, CIE, IMDS*	3
	TC		4	16	20
Manchester Metropolitan University, United Kingdom	TP		1	3	4
	JT		*ITEM*	*ANOR, CS, JCP*	4
	TC		5	29	34
Shenzhen University, China	TP	1	1	2	4
	JT	*TRE*	*TRE*	*IJPR, JCP*	3
	TC	94	29	23	146

Minimum of four articles for institution inclusion. TP = Total Publications. JT = Journal Title. TC = Total Citations. Oct. = October. *ANOR* = *Annals of Operations Research. BSE* = *Business Strategy and the Environment. CIE* = *Computers and Industrial Engineering. COR* = *Computers and Operations Research. CS* = *Computers and Security. IJIM* = *International Journal of Information Management. IJLRA* = *International Journal of Logistics Research and Applications. IJPE* = *International Journal of Production Economics. IJPR* = *International Journal of Production Research. IMDS* = *Industrial Management and Data Systems. ITEM* = *IEEE Transactions on Engineering Management. ITVT* = *IEEE Transactions on Vehicular Technology. TFSC* = *Technological Forecasting and Social Change. TRE* = *Transportation Research Part E: Logistics and Transportation Review*

#### Top contributing countries of blockchain for SSCM research

**Fig. 4:**
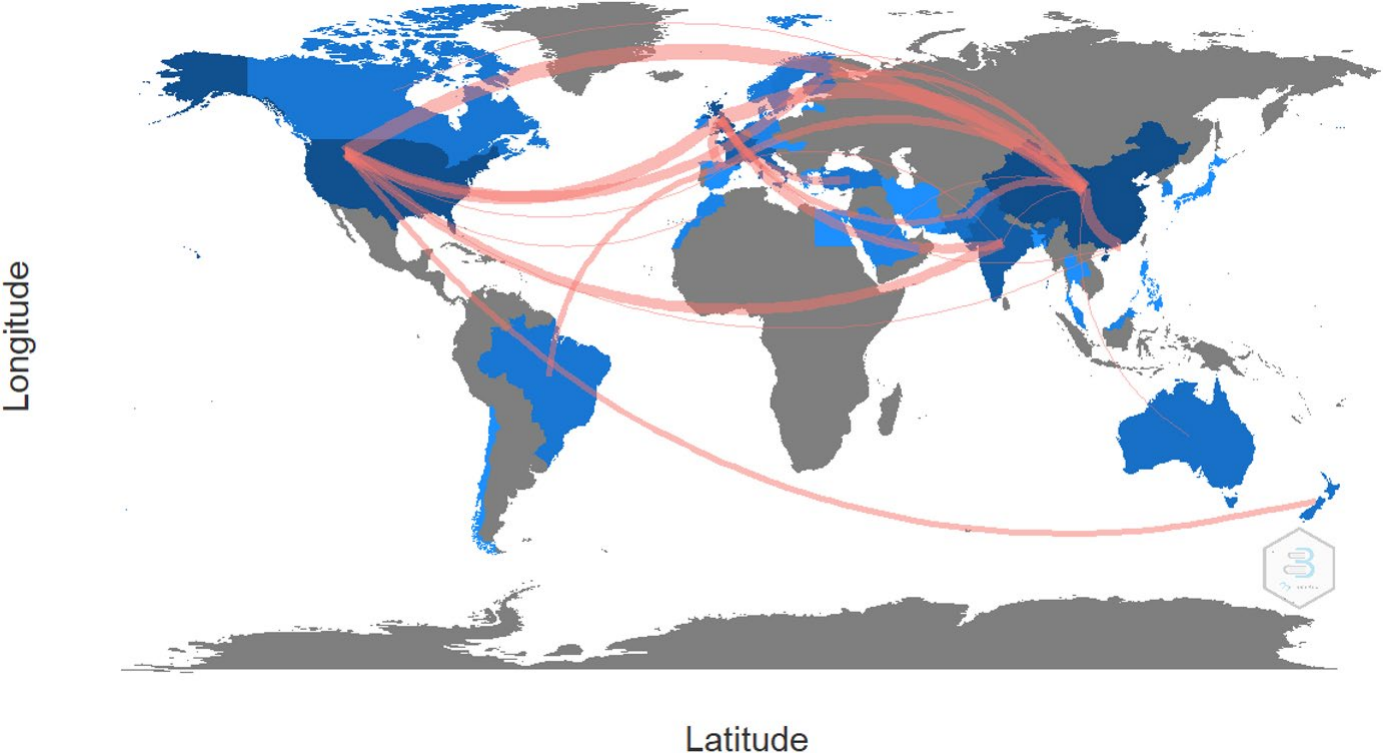
World map of country collaboration on blockchain for SSCM research

An evaluation of country collaboration based on co-authorship is carried out to understand how different countries publishing blockchain for SSCM research in high-quality journals are interconnected, the results of which are summarized in Fig. 4 and Table 7. Specifically, the thickness of the link between two countries in Fig. 4 indicates the degree of co-authorship (collaboration), whereas the specific details of country collaboration (i.e., country of origin of author’s institution) are presented in Table 7. There were 85 pairs of country partnerships identified, with the majority being China and the United Kingdom (11 articles), as well as China and the United States (11 articles), which accounted for 22 of the 146 articles (15.07%). The analysis also reveals that country collaborations between the United States and United Kingdom (*n* = 8), China and Hong Kong (*n* = 7), United States and Finland (*n* = 6), United States and India (*n* = 6), United Kingdom and France (*n* = 5), and United Kingdom and India (*n* = 5), are relatively higher than the other country collaborations. An additional analysis to scrutinize the most-cited countries in Biblioshiny reveals that the countries with the highest research impact and influence are China (TP = 43, TC = 802, C/P = 18.65), United States (TP = 39, TC = 1208, C/P = 30.97), United Kingdom (TP = 36, TC = 669, C/P = 18.58), India (TP = 18, TC = 452, C/P = 25.11), France (TP = 15, TC = 399, C/P = 26.60), Italy (TP = 12, TC = 156, C/P = 13.00), and Hong Kong (TP = 10, TC = 253, C/P = 25.30).

**Table 7:** Country collaboration on blockchain for SSCM research

From	To	*n*	From	To	*n*	From	To	*n*
United States	Australia	1	United Kingdom	Australia	1	China	Australia	2
	Canada	1		Bangladesh	1		Canada	2
	Chile	1		Chile	1		Finland	3
	Finland	6		Denmark	1		France	4
	France	1		France	5		Hong Kong	7
	Greece	1		Greece	1		India	2
	Hong Kong	2		Hong Kong	2		Japan	1
	India	6		India	5		Malaysia	1
	Japan	1		Ireland	1		New Zealand	1
	Morocco	2		Malaysia	1		Norway	2
	Netherlands	1		Morocco	1		Pakistan	4
	New Zealand	3		New Zealand	1		Philippines	1
	Sweden	1		Pakistan	3		Saudi Arabia	2
	Switzerland	2		Philippines	1		Sweden	1
	United Kingdom	8		Qatar	1		Turkey	1
				Sweden	1		United Kingdom	11
				Thailand	1		United States	11
				Turkey	4			
France	Brazil	3	India	Australia	1	Pakistan	Canada	1
	Denmark	1		Brazil	1		Ireland	1
	Egypt	1		France	2		Korea	1
	Germany	1		Korea	1		New Zealand	1
	Italy	1		Morocco	1			
	Korea	1		Netherlands	1	Australia	Canada	1
	Malaysia	1		Switzerland	1		Saudi Arabia	1
	Morocco	1		Turkey	1			
	Pakistan	1				Canada	Ireland	1
	Saudi Arabia	1	New Zealand	Canada	1		Saudi Arabia	1
	Sweden	1		Sweden	1			
	Turkey	1				Sweden	Ireland	1
			Italy	Denmark	1		Switzerland	1
Turkey	Denmark	1		Latvia	1			

*n* = Frequency. Co-authorship structure reflects country collaboration on a pair-by-pair basis

### Science mapping

#### Co-occurrence analysis of blockchain for SSCM research

**Fig. 5:**
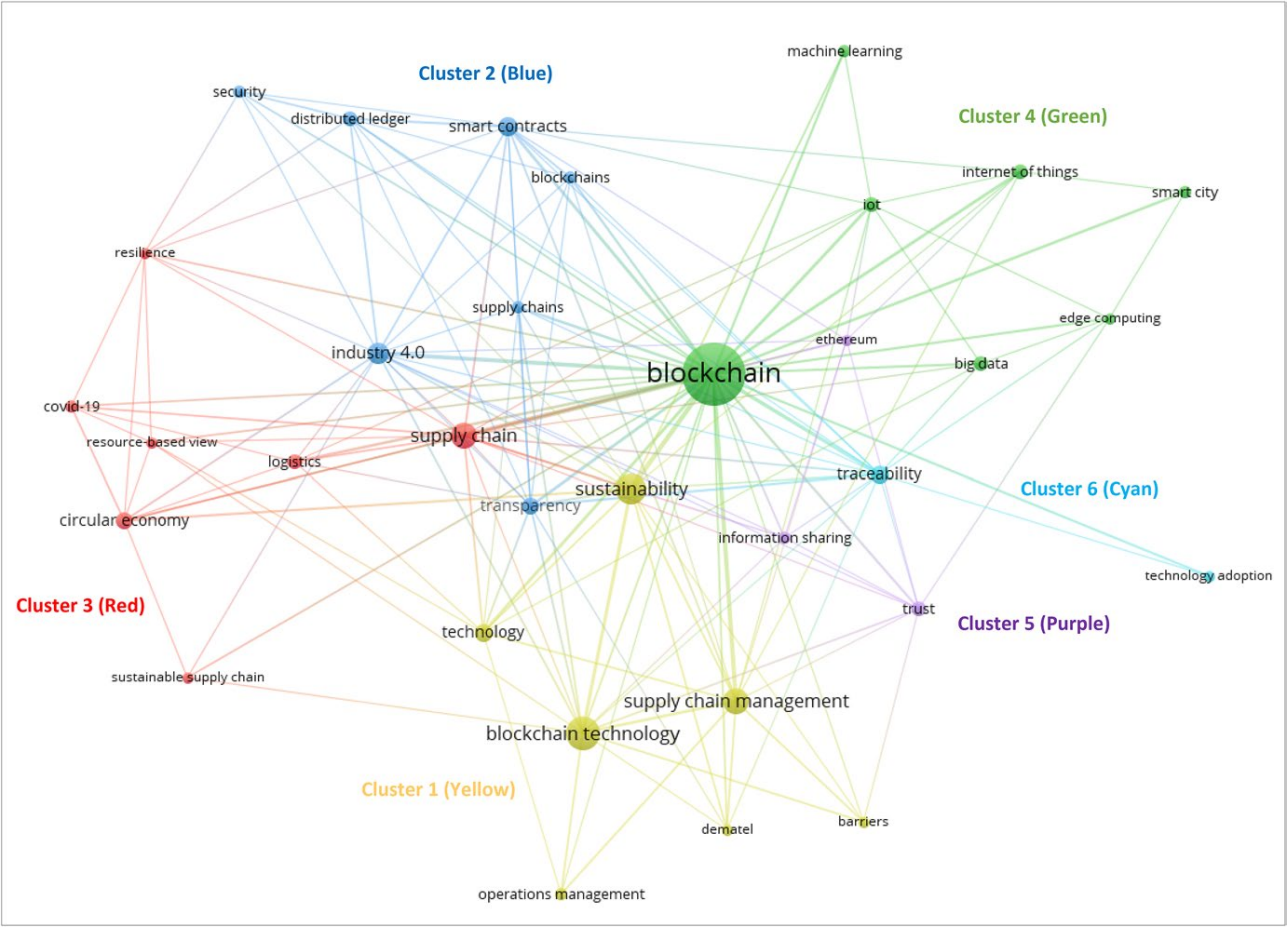
Co-occurrence network of keywords on blockchain for SSCM research

Author keywords are commonly used in a bibliometric analysis technique called co-occurrence analysis to identify commonalities or major themes in a corpus of articles [29, 58, 107]. In this review, the 146 articles in the corpus of blockchain for SSCM research presents 534 author keywords. Co-occurrence of author’s keywords, which refers to the common presence, frequency of recurrence, and near proximity of comparable keywords found throughout the review corpus, is one of the scientific mapping techniques for identifying knowledge clusters [58]. In essence, the co-occurrence in a co-occurrence analysis indicates keywords that are similar to one another and thus related to the same issue, but not identical [58]. The scientometrics tool “co-occurrence analysis” was conducted using the smart local moving algorithm [108] in the VOSviewer software with a minimum occurrence threshold set at three occurrences for keyword inclusion, resulting in a keyword network consisting of 33 author keywords that were automatically segmented into six clusters that represented the major themes in the field of blockchain for SSCM (see Fig. 5). The size of the node representing each keyword in the keyword network indicates the occurrence of that keyword, whereby the larger the node, the greater its occurrence [58]. The thickness of the link between nodes in the keyword network indicates the occurrence that nodes in that link co-occur together, whereby the thicker the link between nodes, the greater the co-occurrence of those nodes [58]. The frequency of occurrence (*n*) and total link strength (TLS) of keywords are presented in Table 8.

**Table 8:** Co-occurrence analysis clustering of keywords

Keywords	*n*	TLS	Keywords	*n*	TLS
*Cluster 1: Blockchain technology and SSCM (Keywords = 7)*			*Cluster 2: Smart contracts and SSCM (Keywords = 7)*		
Barriers	3	7	Blockchains	4	11
Blockchain technology	26	31	Distributed ledger	5	15
Dematel	3	9	Industry 4.0	11	31
Operations management	4	6	Security	4	9
Supply chain management	16	31	Smart contracts	9	25
Sustainability	21	44	Supply chains	4	15
Technology	8	18	Transparency	7	24
*Cluster 3: Circular economy and logistics in SSCM (Keywords = 7)*			*Cluster 4: Blockchain and new-age technological integration in SSCM (Keywords = 7)*		
Circular economy	7	15	Big data	5	8
Covid-19	4	9	Blockchain	89	133
Logistics	5	12	Edge computing	3	8
Resilience	3	11	Internet of things	5	11
Resource-based view	3	8	IoT	5	14
Supply chain	16	39	Machine Learning	4	7
Sustainable supply chain	3	5	Smart City	4	6
*Cluster 5: Ethereum and SSCM (Keywords = 3)*			*Cluster 6: Blockchain and traceability of SSCM (Keywords = 2)*		
Ethereum	3	11	Technology adoption	3	5
Information sharing	4	9	Traceability	8	26
Trust	5	13			

*n* = frequency of occurrence, *TLS* = total link strength

Cluster 1 (yellow) encapsulates seven keywords and concentrates on the “barriers”, “technology”, and techniques (e.g., “dematel”) of “blockchain technology” for “sustainability” in “operations management” and “supply chain management”. The “dematel” or decision-making trial and evaluation laboratory technique is a prominent research method in this cluster, with “blockchain technology” for achieving “sustainability” in “supply chain management” being the lynchpins for research in this cluster. Thus, this cluster exemplifies the theme of *blockchain technology and SSCM*.

Cluster 2 (blue) comprises seven keywords and centers on “Industry 4.0” and “smart contracts” in SSCM. The research in this cluster exhibits the “distributed ledger” aspect of “blockchain” in the form of “smart contracts” as an “Industry 4.0” solution for enhancing “transparency” and “security” of “supply chains”. Thus, this cluster exemplifies the theme of *smart contracts and SSCM*.

Cluster 3 (red) consists of seven keywords and considers how blockchain has been utilized to achieve “resilience” in the “circular economy” and “logistics” in “sustainable supply chain”, including in light of crises such as the “covid-19” pandemic. The “resource-based view” is a prominent theoretical lens in this cluster, with “supply chain”, “circular economy”, and “logistics” being notable lynchpins for research in this cluster. Thus, this cluster exemplifies the theme of *circular economy and logistics in SSCM*.

Cluster 4 (green) contains seven keywords and highlights the emergence and integration of new-age technologies for SSCM through the use of blockchain in conjunction with “edge computing” and “machine learning” to handle “big data” generated by the “internet of things” or “iot” for intelligent operations management in a “smart city”. Thus, this cluster exemplifies the theme of *blockchain and new-age technological integration in SSCM*.

Cluster 5 (purple) incorporates three keywords that shed light on “ethereum” as a blockchain platform and the “trust” that it can foster for “information sharing” in supply chains. Thus, this cluster exemplifies the theme of *ethereum and SSCM*.

Cluster 6 (cyan) is made up of two keywords, focusing on the “traceability” of SSCM activities through the “technology adoption” of blockchain. Thus, this cluster exemplifies the theme of *blockchain and traceability of SSCM*.

#### Factorial analysis of blockchain for SSCM research

**Fig. 6:**
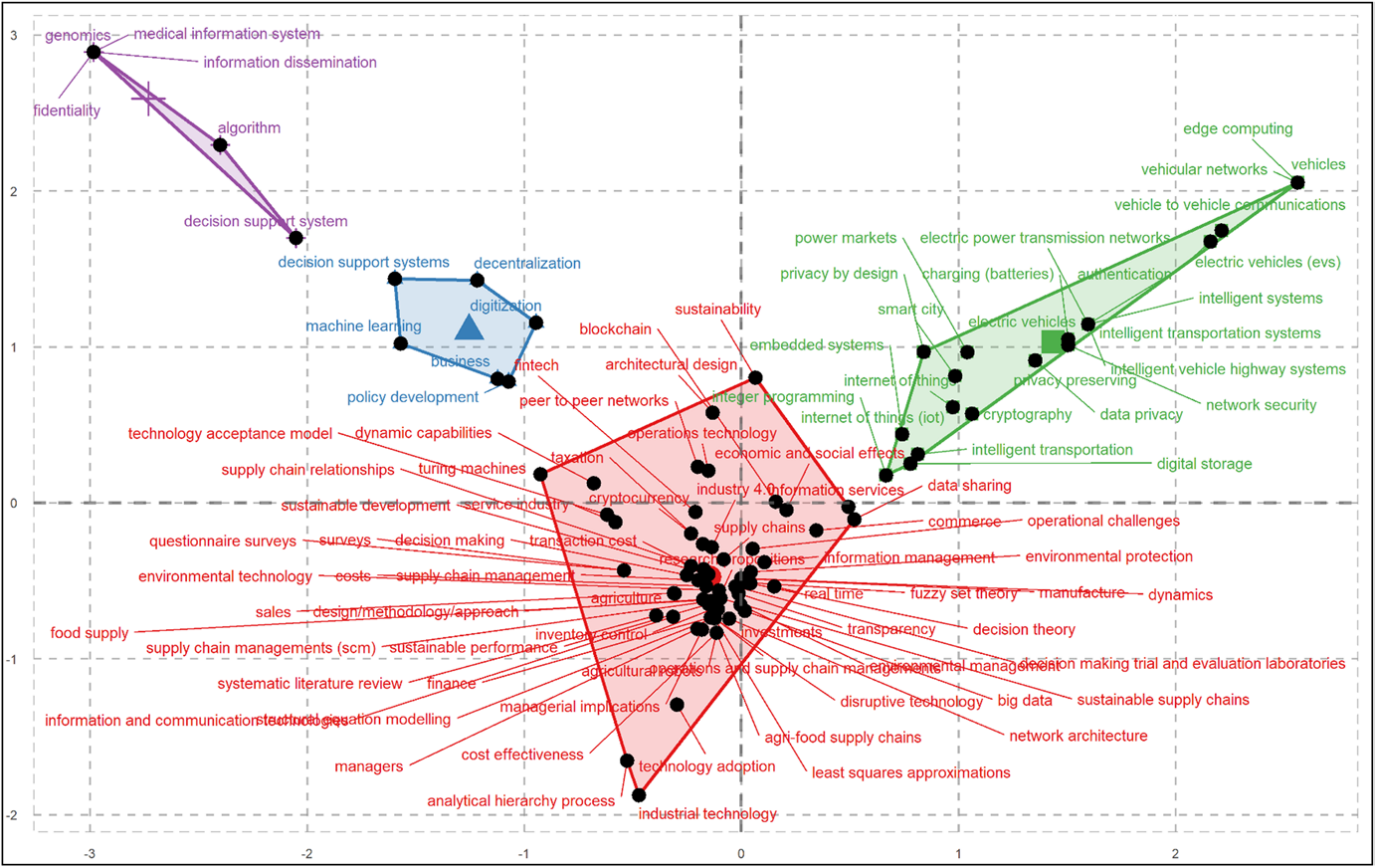
Factorial network of keywords on blockchain for SSCM research

**Fig. 7:**
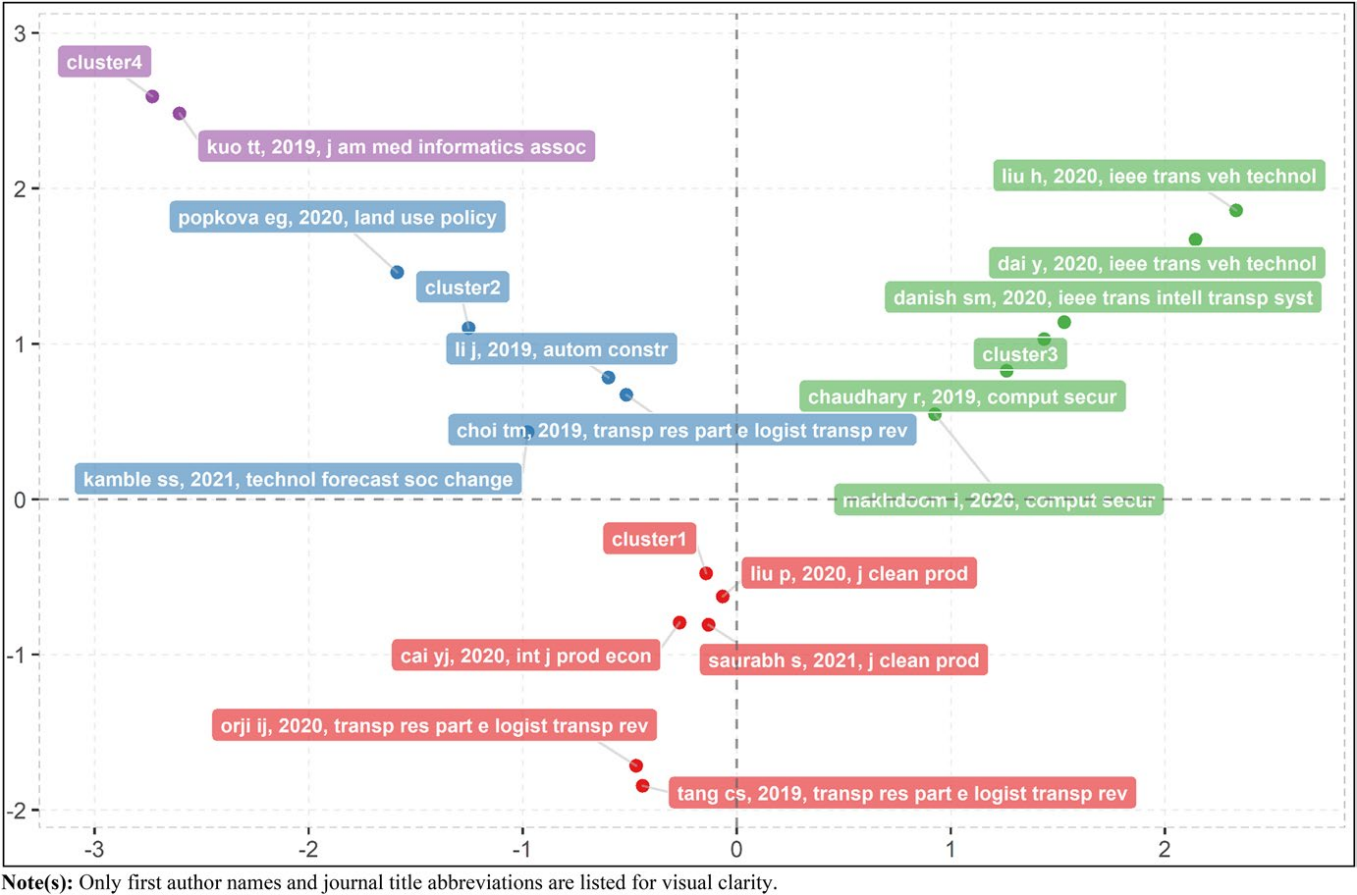
Factorial network of key publications on blockchain for SSCM research. *Note(s)* Only first author names and journal title abbreviations are listed for visual clarity

Factorial analysis is another approach used in scientific mapping, where descriptors and descriptor extraction are employed in topic classification, with descriptors being collections of words that describe the content of a cluster. Biblioshiny for Bibliometrix on R offers an alternate method for investigating the intellectual structure of articles in a specific domain using a technique known as factorial analysis [56]. Factorial analysis is a well-established method in the realm of text mining for the identification of conceptual structure, though it remains underutilized in science mapping [109]. To further understand the intellectual structure of high-quality research on blockchain for SSCM, a factorial analysis is performed using the correspondence analytical technique on all author keywords, which resulted in the classification of four clusters or major themes, as seen in Fig. 6. Correspondence factor analysis is a multivariate approach that may be used on any type of data with any number of data points. It recognizes existing linkages and oppositions between subjects and elements, and it calculates their contribution to total entropy for each factor. Such factorial analysis approach, which was utilized for science mapping, also assisted in the categorization of the corpus articles into four clusters or primary themes. Cluster 1 (Red) is the largest cluster with keywords from 98 articles, whereas Cluster 3 (Green) is the second largest cluster with keywords from 27 articles, and Clusters 2 (Blue) and 4 (Purple) feature keywords from 14 and seven articles, respectively. The articles that were most significant (i.e., contributed the most citations) in each cluster are presented in Fig. 7. Noteworthily, the six clusters identified through the co-occurrence analysis of author keywords with a minimum threshold for keyword inclusion at three occurrences had considerable connection with the four clusters identified in factorial analysis, with its triangulation presented in Table 9. Following the factorial analysis, a review of articles was performed to determine the theme of each cluster, which is summarized in the next sections.

**Table 9:** Triangulation of clusters or major themes of blockchain for SSCM research

Factorial analysis	Co-occurrence analysis
Cluster 1: Blockchain for Sustainable Business Activities	Cluster 1: Blockchain Technology and SSCM Cluster 3: Circular Economy and Logistics in SSCM (Partially Mapped) Cluster 4: Blockchain and New-Age Technological Integration in SSCM (Partially Mapped)
Clusters 2 and 4: Decision Support Systems using Blockchain*	Cluster 2: Smart Contracts and SSCM (Partially Mapped) Cluster 4: Blockchain and New-Age Technological Integration in SSCM (Partially Mapped) Cluster 5: Ethereum and SSCM Cluster 6: Blockchain and Traceability of SSCM
Cluster 3: Blockchain for Intelligent Transportation System	Cluster 3: Circular Economy and Logistics in SSCM (Partially Mapped) Cluster 4: Blockchain and New-Age Technological Integration in SSCM (Partially Mapped)

Though Clusters 2 and 4 manifested separately in the factorial analysis (i.e., the former includes all sectors, whereas the latter focus specifically on the healthcare sector), they are discussed con-currently given the similarity in foundational insights (i.e., decision support systems using blockchain). This treatment is supported by Fig. 6 and Fig. 7, which show the two clusters falling into the same upper-left quadrant of the factorial network

*Cluster 1 (Red): Blockchain for Sustainable Business Activities.* Our commercial activities can produce a negative impact on the earth’s ecosystem (environment and society) in both direct and indirect ways [86]. In this regard, global sustainability initiatives are becoming increasing important for humanity [110, 111]. Fortunately, as documented by the articles in this cluster, there is an abundance of evidences about how business and society are embracing new-age technologies such as blockchain to address the most pressing questions about economic, environmental, and social sustainability [33, 75, 112]. As the planet becomes increasingly connected, firms are now embedding large numbers of IoT devices, smart sensors, and digitized actuators into their product offerings and physical infrastructure [2, 94]. As a result, IoT and blockchain technology convergence is evolving across various sectors, with blockchain applications throughout the SC operating with data generated automatically from physical IoT devices in the business ecosystem, as suggested by the articles in this cluster [50, 110, 113].

With consumers demanding greater transparency and increasing complexities of SSCM, the articles in this cluster also indicate that blockchain is a simple and affordable solution to track each material used in the finished product, which is crucial to building trust with increasingly environmentally- and socially-conscious consumers [57, 80, 111]. Increasing the visibility of materials throughout the SC with blockchain aids in the authentication against counterfeit products and reduces administrative costs for all SC partners [12, 114]. Therefore, blockchain is said to be playing an evolving role in supporting sustainability in SC activities across sectors (e.g., food, healthcare, infrastructure, manufacturing, and retail sectors) by cultivating mutual trust and collaboration across all stakeholders in a multi-echelon SC [44], aiding customers to adopt sustainable lifestyles [13], and assisting firms to strengthen their procurement and recycling processes [8].

Though evidences of blockchain application across different sectors may vary in motivation and appear dispersed, the large majority of articles in this cluster remain in the reasoning phase, or in other words, a heavy reliance on argument and logic to clarify the value, present state, barriers, and prospects of blockchain for SSCM [9, 22, 88, 115]. This may due to the fact that blockchain technologies are in early trials in the global SC network, as evidenced by ongoing discussions of studies conducted in both developed and developing economies [46, 72]. Most firms are still learning about the cost, security, and implications of introducing a blockchain-based framework for managing sustainability in the SC, with many firms finding connecting blockchain to physical objects as a major operational challenge [15, 19, 39, 114]. This is exacerbated by the challenge that a focal firm faces in convincing its partnering stakeholders to embrace blockchain for greater sustainability in the SC [1].

Finally, this cluster also highlights the emerging concept of the circular economy and its three foundational R’s that are crucial for SSCM: (1) *reduce* materials and waste, (2) *reuse* products, and (3) *recycle* materials. The use of blockchain technology in such a paradigm enables materials and products to be traded in closed processes or cyclic stages, ensuring that nothing goes to waste and thus empowering SC members to conserve natural resources more effectively [33, 71]. Further discussion and evidences of blockchain use for SSCM across various sectors avail in the later sections.

*Clusters 2 (Blue) and 4 (Purple): Decision Support Systems using Blockchain.* Decision support systems (DSS) are widely regarded as a subset of business intelligence that aims to assist firms in making informed business decisions based on information derived from massive amounts of analyzed data via techniques such as data warehousing, data mining, and data analytics [31, 85]. Data analytics for firms is advancing at an exponential rate with each passing year and is now associated with smart business paradigms associated with new-age technologies such as artificial intelligence and machine learning [116].

In particular, the articles in the second and fourth clusters have highlighted the convergence between machine learning and blockchain technologies, with the second cluster focusing on decision support systems predicated on this convergence across sectors[20, 50, 114], and the fourth cluster specifying the same specifically for the healthcare [85]. Machine learning, which is well-known for its ability to process massive amounts of data, focuses on developing sophisticated algorithms that can learn and optimize themselves based on prior experience (without being explicitly programmed), and thus, making remarkable developments for effective decision making through identifying trends and drawing insights from factual evidence [116]. The convergence of machine learning with blockchain offers the opportunity of developing improved predictive algorithms through blockchain’s decentralized architecture for achieving sustainability in SC [31].

The articles in the second cluster also demonstrate how the convergence of blockchain and machine learning are reducing end-to-end SC problems in agriculture by allowing traceability of food supplies throughout the SC and managing financial transactions between SC partners [9, 20, 93, 117, 118]. For example, in collaboration with Nairobi-based Twiga Foods, IBM unveiled a blockchain-based microfinancing strategy for food vendors [119]. IBM researchers studied vendor transaction records and then used a machine learning algorithm to determine their credit worthiness, offering third party lenders the trust they needed to offer microloans to small businesses. Following the determination of the credit score, the established lending network used blockchain based on the hyperledger framework to manage the entire lending process, from loan application to contract offer to agreement on repayment terms. Likewise, IBM’s developed blockchain-based technology has been taken into account by other food companies, like Unilever and Nestlé, to cope with agricultural/food disasters such as waste and pollution. Other articles in this cluster have also shed light on how blockchain can co-exist with other new-age technologies such as drones, RFID, and GPS that feed into machine learning for crop protection, weed detection in farmland, livestock management, crop quality management, site-specific nutrient management, and harvesting [20, 28, 120].

Finally, the articles in the combined cluster also exemplify the epithet “digital economy” [121]. Middlemen wreak havoc on all business sectors around the world, complicating transactions and raising the cost of doing business [111]. The combined cluster provides evidence on how decision support systems for firms built with blockchain and machine learning can disrupt traditional business paradigms and forge their entry into the digital economy by facilitating a peer-to-peer infrastructure model for all SC members, eliminating the need for middlemen [65, 115]. Noteworthily, firms benefit from decision support systems that draw insights from predictive algorithms developed using machine learning approaches to reduce the carbon footprint of SC operations, which can be combined with blockchain technology to ensure transparency and traceability among SC partners across sectors [12, 22].

*Cluster 3 (Green): Blockchain for Intelligent Transportation Systems.* The term “smart cities” refers to a futuristic urban model that uses ICT to assist citizens, communities, and organizations (both public and private) in generating and exchanging real-time data for improving the quality of city operations in areas such as energy use, logistics, and other public services [122]. Intelligent transportation system (ITS) is an essential element for all project ideas of smart cities transmuting existing into modern communities, making urban infrastructure easier for its inhabitants in all aspects [66, 123, 124]. An ITS is a technological platform based on the evolving economic model of the “shared economy,” with the aim of providing customized solutions related to various modes of transportation, traffic, and logistics [13, 125, 126].

The majority of articles in this cluster have advocated for the use of electric vehicles (EVs) to meet global sustainability targets [66, 87, 127–129]. The use of blockchain for EVs and charging infrastructure is a hot topic in this cluster, with the goal of developing a mechanism that ensures the execution of various energy charging scenarios for EVs while meeting requirements for reliability, privacy, and cost [87, 92, 127–129]. These articles have explained various energy charging solutions developed on the blockchain architecture, as well as how an EV using a secure vehicle-to-vehicle communication technology would optimally select the best-suited charging station from the bidding list based on factors such as the scheduled route, the vehicle’s battery level, real-time traffic information, and driver preferences.

Another collection of articles in this cluster proposed various secured data processing and sharing schemes to eliminate malicious intent and social disruptions in order to protect the massive volume of data generated by vehicular social networks [115, 126, 130, 131]. Besides that, articles in this cluster show that urban mobility necessitates the development of more cost-effective and environmentally friendly transportation systems for both public transportation and freight logistics [54, 89, 132]. To retain these sustainable initiatives in urban scenarios, a centralized smart city governance structure should be developed that provides citizens with a blockchain-based incentive scheme for green energy use in EV and ITS extension with smart grid infrastructure, saving them resources, money, and time while making their city smarter on all dimensions of the triple bottom line of people, planet, and profit [66, 122].

#### Content analysis of sectors in blockchain for SSCM research

A content analysis was carried out to shed finer-grained insights relating to blockchain for SSCM research in high-quality journals from a sectorial perspective. The 146 articles in the review corpus were meticulously studied to determine the application of blockchain technology across sectors. Noteworthily, sector-specific information cannot be organically determined through a bibliometric analysis, thereby warranting the use of a content analysis. Forward and backward screening was utilized to identify sectors and the articles that fall within each sector-specific cluster, which were collectively discussed and agreed upon by the research team. As a result, four distinct sectors were identified, namely food, healthcare, manufacturing, and infrastructure. Let us take the food sector as an example of a sector identified through the content analysis. We first returned to the Scopus database, where our screened 146 articles were saved, and we then applied filters on the 534 author keywords in the review corpus, where we picked out food sector-related keywords such as “food supply”, “food supply chains”, “agri-food supply chains”, “agriculture”, “agricultural robots”, “agriculture supply chain”, and “agricultural supply chains”, resulting in a total of 15 (out of 146) articles related to the food sector. Following that, the identified articles were reviewed to ascertain if an explicit discussion on the food sector exist, and if so, to outline their specified application. The other three sectors were also identified and scrutinized in a similar manner. In this regard, the forward and backward screening served as a useful cross-check mechanism to ensure the trustworthiness of the content analysis. The insights for each sector are summarized in the next sections.

*The Food Sector.* The SC in the food sector is diverse and international, with raw material suppliers and indigenous product sources spanning across the globe [90, 111, 120], which makes tracking the movement of commodities and raw materials from farmers to end users and maintaining traceability through the SC highly challenging [9, 31, 115].

In recent times, food recalls are omnipresent, with multiple products being recalled everyday due to sub-standard quality and health concerns, raising concerns among stakeholders in the food SC, with the primary trigger being the unwillingness of SC partners to adhere to sound manufacturing standards [12, 20]. In this regard, blockchain has enormous potential to resolve such issues in the food sector by connecting the physical and digital realms via real-time capturing of information such as temperature, humidity, and movement on a digital secured ledger accessible to all SC members during product shipment or storage for non-compliance notifications while ensuring no data tampering [2, 9, 31, 44]. This cutting-edge remote monitoring technology can help focal firms to keep abreast of compliance, promote accountability among SC members, and take corrective measures for quality control.

The benefits of blockchain are applicable not only to the food SC for agricultural produce, but also animal husbandry and fisheries [39, 86]. In particular, blockchain implementation can be beneficial to SC in the food sector, especially when combined with genome codification and nutrient sampling reports [12, 18, 111]. Additionally, the usage of a Quick Response (QR) code or a Radio Frequency Identification (RFID) tag on an end product can include details about the source of specific components or information on manufacturing conditions and distribution, allowing end users to leverage the benefits of traceability and transparency [18, 28, 86]. Such traceable and reliable information is therefore essential for all parties involved within the food SC to ensure compliance with food regulation and documentation [111, 120]. Furthermore, understanding the origin of a food commodity, as well as other additional information, has a major influence on a consumer’s buying decision, especially among consumers who subscribe to the values of sustainable consumption [75, 90].

Apart from economic benefits for all SC members, the benefits of blockchain implementation also extends to other facets of sustainability gains, such as reducing climate change and environmental degradation and safeguarding of human rights and social welfare. On the environmental sustainability aspect, blockchain-based IoT solutions are being proposed for the monitoring of many activities such as crop quality management [20], illegal/over-fishing [12], livestock health management [20], and conservation project funding [86]. On the social sustainability aspect, firms participating in the food SC face the daunting challenge of ensuring that no unacceptable labor practices or activities related to human rights violations occurred in the SC [2, 39, 75]. Firms can deploy a smart contract system powered by blockchain to exchange employee details such as compensation terms, actual work performance, contract period, and labor conditions with all members of the upstream and downstream SC [133]. This can also minimize the number of intermediaries in the SC, thereby lowering transaction costs, improving profit margins, and transferring a significant portion of income to the farmer/producer. Crop protection insurance is also another socioeconomic contribution of blockchain technology in the food SC, providing farmers with economic and social security in the event of unanticipated crop loss, as well as with the ability to detect and avoid fraudulent activities [39].

*The Healthcare Sector.* Blockchain supports the pharmaceutical SC in the healthcare sector by managing clinical trials and drug (or medicine) inventory while reducing counterfeiting and theft issues [39, 67, 72]. Specifically, drug shortages can cause patients to receive delayed treatment, posing a serious danger to public health [134]. In addition, drug shortages can open doors for counterfeit drugs to enter the market, which poses a danger to healthcare systems [39, 135]. Therefore, the problem of drug scarcity necessitates collaboration among all stakeholders in the healthcare SC so that patients have appropriate and timely access to drugs, wherein blockchain can play an important role by improving the monitoring of market demand for drugs [74, 91].

Drug recall in the healthcare SC is another major problem that can jeopardize the economic survival of pharmaceutical firms as well as the wellbeing of end users [136]. With the use of digitized transactions, the interoperability nature of a blockchain can provide a simple visualization of the trajectory of a drug lifecycle from manufacturer to patient, allowing SC members to examine vulnerable points in the SC and reduce the chances of fraud and the costs associated with it [67]. Serialization, for example, is a method of labeling drugs that can provide real-time access to multiple stakeholders involved in the drug development process throughout the SC, allowing them to meet the Food and Drug Administration’s (FDA) stringent requirements [57, 136]. This also ensures that the source of drugs are authenticated using a blockchain-based serialization mechanism, mitigating the risk of counterfeit or duplicate drugs in the SC [82].

Noteworthily, the continuous monitoring functionality, which allows each stakeholder (e.g., suppliers, manufacturers, wholesalers, retailers, pharmacies, hospitals, and consumers) to record each transaction on a distributed ledger, barcode, or QR code on drug packaging authenticated at all levels in the SC using a blockchain platform can prevent the market penetration of counterfeit drugs [82]. This ensures that drug orders in the healthcare SC are completed on time and without errors. Furthermore, the permissioned ledger nature of blockchain can reduce labeling inefficiency (e.g., misleading or false information) of a non-compliant member in the healthcare SC and facilitate efficient recall management by allowing the identification of exact locations of drugs. Finally, another critical application of blockchain in the healthcare sector is its facilitation of recycling activities of pharmaceutical products as well as its mitigation of carbon footprints of pharmaceutical firms, raw-material suppliers, and other member firms, thereby allowing the government to better predict carbon taxes for each firm in order to account, and hopefully reduce, their negative impact on the environment [19].

*The Manufacturing Sector.* The SC for commodities manufactured by a focal manufacturer is often highly complex, with the upstream channel consisting of several tiers of strategic suppliers scattered globally and the downstream channel consisting of several tiers of distributors whose role is to ensure that every product manufactured by a focal manufacturer reaches the end user [2, 3, 5]. In this regard, there is an increasing need to simplify operational complexities in the manufacturing SC, which can occur by improving transparency and traceability among SC participants, as well as by reducing operational disruptions in the SC [24, 46, 110]. Since manufacturing necessitates ongoing communication among SC partners, a single data breach or malpractice can jeopardize each SC partner’s manufacturing operations. Validating transactions between SC members in such a complex SC often takes a long time and can be stressful due to the inherent complexity of SC members’ diverse geographic locations and the numerous transactions related to buying and selling of commodities or raw materials and semi-finished and finished products throughout the SC [4, 77]. In such scenarios, blockchain can act as an interoperable lock box for authenticating commodity or raw material sourcing across the upstream SC and certifying supplier parts to ensure consistency in manufacturing of quality products [1, 8, 80]. This practice can be useful in detecting any malfunction or quality failure when the product is in use, as well as in cases of subsequent recalls to track back quality issues and the accountable source [18].

Furthermore, manufacturers are using IoT to monitor production activities within their upstream SC to assess real-world working conditions of their suppliers and their conformance with social sustainability activities enforced by the focal manufacturer while keeping regulatory requirements in check [2, 8, 16]. Unplanned maintenance of a supplier in the SC can trigger machinery downtime and distribution delays of raw material supply, undermining the production operations of a focal manufacturer, resulting in severe economic repercussions. In this regard, blockchain can be used in conjunction with IoT to track and optimize the operations of supplier maintenance and inventory management activities to reduce the lead time of raw material supply [20, 124].

Moreover, decentralizing production activities in the upstream SC through additive manufacturing (also known as 3D printing) can offer numerous benefits to the focal manufacturer and its suppliers (e.g., reduced design and logistic costs, reduced lead time), but these benefits can only be realized with interorganizational trust and secured SC network [16, 22, 39]. Successfully deploying additive manufacturing across upstream SC is more of a database or record management challenge than a functional or equipment integration issue [137]. The distributed additive manufacturing SC is rendered possible by blockchain-based digital thread, which is considered the backbone of digitized SC that carries 3D-printing information to concerned SC participants for the relative simplicity of printing components on-site [22, 137]. Indeed, additive manufacturing has enormous potential for reducing the need for energy-intensive and logistically-reliant operations, reducing the resources needed in the SC and enabling more socially sustainable activities.

In the downstream manufacturing SC, an increasing number of distributors (e.g., wholesalers and retailers) are eager to use cutting-edge blockchain and IoT technologies to address a variety of issues, including end-to-end SC visibility, anti-counterfeiting, quick product recall, reverse logistics of returned products, certifying reliable suppliers, and secured platform for payments [18, 50, 57, 88, 138, 139]. Therefore, the adoption of blockchain in the downstream manufacturing SC can reduce SC risk by preventing fraud and counterfeiting.

Noteworthily, blockchain-enabled IoT technology in a manufacturing SC can provide all SC parties with secure, seamless, and on-demand visibility over cargo movement, which can result in benefits such as reduced payment time, accelerated delivery, improved sales, and cost savings through tracking authenticated data such as freight volume, traffic congestion, and accidents [49, 50, 54, 57]. Similarly, the use of blockchain technologies for freight monitoring can lead to quicker resolution of insurance claims in situations when cargo is missing or damaged [48, 125].

*The Infrastructure Sector.* Combating the complexities of rapid urbanization requires innovations and scenario planning that challenges traditional models of city development [122, 123, 140]. The idea of a “smarter city” has become habitable, prosperous, and productive for urban netizens with the use of new technological platforms that have the ability to fundamentally transform the way people, organizations, and governments engage with one another [13, 124]. Blockchain is one such groundbreaking new-age technology that will help cities become smarter [11], with developments in the built environment [13, 45], urban mobility [54, 92], and energy networks [66, 73, 87] playing critical roles.

The research community is actively exploring the use of blockchain technologies for SSCM in the built environment and construction sector, which is still in its early stages [81, 141]. A notarized mechanism for verifying building records in accordance with regulatory standards, as well as an authentication mechanism that allows metropolitan builders to automatically obtain building materials from suppliers and process payments to them, are examples of application areas [57, 94]. Furthermore, in conjunction with building information modeling (BIM) (software-based), IoT-enabled remote monitoring, and visualization dashboards, blockchain-based program architecture can be used to manage and monitor processes (e.g., life-cycle expenses, tenancy habits, operational energy analysis, water consumption, interior climatization, waste disposal) within building structures for quantifying carbon footprint and improving human comfort [94, 141].

Moreover, blockchain technology can be deployed to create a marketplace for electric power supply [66, 127]. Microgeneration of electricity using rooftop photo-voltaic solar panels can replace conventional energy sources and encourage the usage of renewable energy sources [73, 84]. Specifically, a record of electricity generated and utilized by each user in the grid can be recorded on a blockchain using smart meters, with incentives (or credits) distributed to the user for surplus power supply as well as credits repaid for power consumption [73, 123]. This essentially offers a peer-to-peer trading mechanism in the infrastructure sector for energy supply networks in a way that is transparent, hassle-free, and reliable [66].

## Gaps and suggestions for future research

The corpus of articles on blockchain for SSCM research published in high-quality journals spreads across diverse areas (themes, scopes). Using a content analysis, this paper identifies noteworthy gaps and suggestions for future research for blockchain application in SSCM in general and across specific sectors, which will be discussed in the next sections and summarized in Table 10.

**Table 10:** Future research agenda on blockchain for SSCM research

Sector	Future research questions
General SC	▪ What frameworks can be used to determine the feasibility of deploying blockchain technology for SSCM across sectors [41, 76]? ▪ What are the types of metrics and information sources in different blockchain systems that can be used for SSCM? [113] ▪ How has the degree of blockchain adoption impacted the productivity, visibility, and traceability of sustainability operations within SC over the years [1, 14, 46, 51, 79]? ▪ How have the challenges to blockchain technology adoption for SSCM evolved over the years [1, 19, 72]? ▪ What are the factors that influence the adoption of blockchain for SSCM in extraneous contexts (e.g., diverse geographical locations, differing industry settings) [53, 70, 89, 112]? ▪ How can social media analytics enable blockchain technology to support sustainable operations in the sharing economy model and rental service platforms [25, 114]? ▪ What are the emerging blockchain-based mechanisms for funding enterprise startups (or new eco-projects) and administrating omnichannel retail for sustainable products [65, 88, 142]? ▪ What is the implementation viability of adopting blockchain technology across SC in terms of standardization, speed of operation, and transaction cost [40, 55, 65]? I▪ n terms of providing significant insight on sustainable performance, how does the financial reporting standard of companies embracing blockchain vary from that of non-adopters [17]? ▪ How do external stakeholders such as government regulators and non-governmental organizations (NGOs) monitor and inspect sustainable SC activities [1]? ▪ What are the auditing mechanisms that could be developed for blockchain-based business processes across sectors [75, 91]? ▪ How can blockchain technologies be embedded into enterprise resource planning (ERP) to provide a highly secure collaboration platform where records of operational activities (compliant or non-compliant) can be conveniently exchanged among trusted parties [9, 20, 115]? ▪ How can machine learning algorithms assist blockchain-enabled ERP systems in optimizing resource utilization and productivity to achieve sustainability in SC activities [20, 31]? ▪ What are the regulatory implications for technological scalability of blockchain for SSCM [17]? ▪ For sustainable SC members interested in adopting blockchain services, how could the adoption choices of other members influence their own blockchain adoption behavior? [139]
Food SC	▪ What are the enablers for implementing blockchain in the food SC (e.g., agriculture, fishery) in emerging economies with poor technology penetration [90, 111, 143]? ▪ What is the return on investment for blockchain adoption and technological scalability in implementing a multi-stage visualization and traceability framework across the food SC [9]? ▪ How will the long-term effects (or triple bottom line benefits) of adopting sustainable activities in the food SC using blockchain technologies be assessed or audited [12, 44]? ▪ What policy mechanisms should be developed or replicated through which regulatory authorities (or governments) can advocate for blockchain-based procurement mechanisms in the food SC to help farmers/fishermen/food producers ensure their social and economic security while also guaranteeing food safety for consumers [20]? ▪ How should regulatory authorities (or governments) curate subsidies (or incentives) for capital investments, environmental protection programs, workforce training, and SC member education in the food sector through various policy measures, and how can SC partners advocate for such policies [20, 86, 143]? ▪ What is the impact of blockchain technology on social sustainability concerns, particularly the legal and ethical implications of its use in food SC [6]? ▪ In an agricultural SC with unpredictable yield, under what circumstances will the retailer adopt blockchain? How does the reluctance of a SC member to participate in an agribusiness SC affect the procurement process as well as the firm’s competitiveness [118]? ▪ How can blockchain challenges and practices experienced in Halal food SC be prioritized and ranked [26]?
Healthcare SC	▪ How can the technical architecture of a blockchain-based system be designed to efficiently handle a massive volume of patient data across multiple service touchpoints for patient health and care [67, 85, 136]? ▪ What procedures should be followed in a blockchain-based identity verification system in the healthcare SC in an emergency scenario where medical professionals (e.g., doctors, physicians) can access patient information without authorization and gather data on the patient’s medical history [67]? ▪ How can blockchain-based smart contracts be used to automate the procurement, distribution, and inventory management of medical supplies in the healthcare SC in a cost-effective manner [136]? ▪ How can the uncertainty of medical supplies in the healthcare SC be minimized and addressed using blockchain-enabled predictive modeling algorithms across emergency scenarios (e.g., supply breakdown, epidemic outbreak, natural catastrophe) [134, 136]? ▪ How can healthcare SC reconcile greater sales through multiple channels with the cost of information sharing related to blockchain [144] ?
Manufacturing SC	▪ How does the disparity in blockchain adoption for SSCM vary across countries as a result of their technical progress [21, 55]? ▪ How do firms teaming up in a manufacturing SC collaborate on a blockchain-based platform for research and development activities and new product design with an emphasis on sustainability [77]? ▪ How do data consistency issues (e.g., quality, confidentiality) affect the long-term viability (or sustainability) of a multi-tier SC operation, and how could a blockchain-based approach address such issues in a manufacturing SC [10, 17, 42]? ▪ What are the unique ways that manufacturers across a diverse operating sectors use blockchain-based digitalization to engage and incentivize customers to reuse and recycle to boost SC sustainability [110]? ▪ How can a policy and legislative framework be developed to support the use of blockchain in the circular economy [33]? ▪ How can blockchain promote innovation (or new product development) in IR4.0, and to what extent can the incorporation of collaborators and processes across the product lifecycle via blockchain support the development of smart and sustainable production facilities [27] ?
Infrastructure SC	▪ How can developing countries with infrastructural constraints build circular economies with the aid of blockchain technology [33, 71]? ▪ How can functional modules in a construction SC be rendered to securely communicate with multiple users and smart connected systems using blockchain to make it more resilient to sustainability [13, 45, 94, 123]? ▪ What are the various blockchain-based approaches for tracing and recycling construction and demolition wastes across the SC to increase its utility longevity and resource efficiency [94]? ▪ What are the numerous technological challenges associated with the scalability of a blockchain-based peer-to-peer trading platform for sustainable opportunities in the urban infrastructure sector [66, 73]? ▪ Are airports and (their) other SC participants conscious of the effects of blockchain technologies for greater sustainability, and what services can airports provide to their travelers that are supported by blockchain technologies to promote sustainable practices [11]? ▪ How are diverse blockchain-enabled governance mechanisms developed with a visualization technology framework for sustainable operation management across various cities adapting to a smart city model [122]? ▪ How can the time taken to reach a consensus in intelligent transportation infrastructure for users of autonomous (or self-driving) cars be reduced [124, 126, 130]? ▪ What are the best practices for collecting and processing relevant data using blockchain in a smart-city charging infrastructure for electric vehicles in a joint operation among energy companies, incentivizing consumers, and informing users on the availability of charging facilities [52, 87, 92, 127, 130, 131]? ▪ How can blockchain improve environmentally-friendly vehicle charging services (e.g., customer satisfaction, share private charging piles) [128]? ▪ How can the planning of intelligent transposition services for cities be improved using blockchain in tandem with last-mile delivery, marketing, integrated mobility, and surveillance [140]?

### Future research agenda for SSCM

Blockchain-based digitization seeks to transform an open SC into a closed-loop SC through which social and ecological concerns are incorporated into business activities [81, 110]. This transformation results in the development of an evolving business ecosystem that assures compliance monitoring, quality assurance, and active incentivization, thereby motivating future research in three directions, namely the circular economy, the digital economy, and the sharing economy, to resolve issues associated with the changing complexities of a closed-loop SC, such as disagreements regarding the adoption of new practices among SC members at various levels [3–5].

To begin, the concept of a circular economy can be described by the 6R model, which includes *rethinking* strategies, *reducing* material consumption and waste, *reusing* products, *recycling* materials, and *repairing* and *repurposing* products and equipment [33, 81, 145]. To help accelerate sustainable development initiatives, effective implementation of the 6R model requires behavioral consensus among stakeholders (e.g., authorities, consumers, business rivals, non-governmental organizations) rather than just technological consensus via the blockchain platform [5, 14, 139]. The unrivaled scalability of blockchain technology is largely inhibited by behavioral disparity and conflict between the participants of the SC [2, 46]. Therefore, future research should focus on establishing social mechanisms centered on policy formulation, sensitization programs, a mutual collaborative working forum, an incentive-for-performance structure, as well as a penalty structure for non-compliant activities to strengthen the interorganizational relationship in the circular economy model using blockchain technology [9, 75, 91].

The circular economy model’s proposed solutions also open up a window of research opportunities for the digital economy model [55, 121]. Several studies, however, suggest that the immaturity of a country’s technological infrastructure inhibits widespread adoption of blockchain technologies in SC [71, 111]. This implies that firms willing to incorporate blockchain into their business operations in such economies have no persuasion control over their SC partners [33, 143]. This provides the opportunity for researchers to study and assess the applicability and value of blockchain technologies for SC in emerging countries and to demonstrate the success of pilot projects to encourage large-scale adoption. In such scenarios, data safety and security could manifest as macro-level challenges [72, 137]. Therefore, future research should also pursue a qualitative approach to identify diverse enablers for successful system deployment utilizing blockchain for SSCM in the context of an emerging economy. To add value in this context, researchers can propose and develop a multitude of maturity model frameworks to assess a firm’s readiness to implement blockchain technology in their SC operations, premised on an inclusive investigational scope of diverse sectors. Furthermore, opportunities for investigation into how information systems in organizations might employ blockchain technology in optimizing resources to achieve sustainability in SC operations have been recommended [20, 90]. Longitudinal studies are needed to determine the degree to which adoption of blockchain technologies affects the performance of sustainability activities inside SCs [1, 14, 46, 72].

Lastly, sustainability in SC operations is also associated with the sharing economy model, and thus, additional research may be conducted to establish new organizational governance mechanisms for sharing resources among SC participants to achieve mutually agreed-upon sustainable goals [20, 75, 115]. As customers have started demanding greater visibility into the SCs of the products they purchase, researchers have proposed investigating how organizations, using social media channels such as Facebook, LinkedIn, and Twitter, are improving their two-way communication with customers and the sharing of information with stakeholders [25, 114]. As this strategy is expected to enhance brand reputation and customer satisfaction, further research is needed to uncover the extent of its impact.

Given the origins of blockchain technology in the financial arena, future research are advised to focus on themes such as financing methods for eco-startups [65], managing sustainability in omni-channel retailing [88], and auditing of sustainable operations [84] given that blockchain technology has the potential to be utilized as a source of automated audit verification procedures [146]. That is to say, instead of requesting bank statements or submitting confirmation requests to the relevant parties, auditors may check transactions on publicly accessible blockchain ledgers. In this regard, researchers could explore the accelerators that can contribute to the effectiveness of automated audit verification techniques in a multitude of sectors. As the sharing economy notion for establishing transparent SCs remains nascent, further research is required to understand how disclosing financial data of companies using blockchain affects their overall performance [17].

### Future research agenda for food SC

Research questions proposed for potential studies in the food SC recognize many obstacles to the deployment of blockchain technology in the sector [118]. To begin, the food SC is comprised of many farmers and fishermen as primary providers who may lack adequate skills to ensure blockchain-based interoperability among SC participants [135]. Further feasibility studies should be conducted to determine how a focal firm could develop a training curriculum and an incentive mechanism for such primary suppliers to potentially increase the rate of blockchain technology adoption in the food SC [139, 146]. Furthermore, the lack of a unified procedural guideline at all levels of the food SC is a major impediment to blockchain adoption [9, 44]. This necessitates additional research by academic scholars to advise the sector’s policymakers and professionals on the formulation of operating procedures and regulations for standardizing institutional metrics relevant to sustainability and supervision of end-to-end SC operations. Moreover, the use of proof of work algorithms require significant technological resources to execute transactions among SC members, indicating that the capital cost of blockchain technology is substantial, which is another cause for SC member reluctance to adopt that technology [1, 40]. Therefore, future research can be conducted to demonstrate empirically, using a case study method or a longitudinal research design, how blockchain implementation will serve as a primary source of cost savings for each member of the food SC. Moreover, prior studies focusing on the food sector, as shown in Table 10, have proposed unique avenues to be explored such as areas with low technical penetration [111, 143], regulatory mechanisms that account for the economic and social interest of all stakeholders [20], and the adoption behavior of SC members under uncertain yield [118], among others.

### Future research agenda for healthcare SC

In the healthcare business, several sources of metadata include hospital records, patient medical records, results of health screenings, and internet of things device records. Furthermore, biomedical research creates a considerable number of metadata important to public healthcare. Interested researchers might look into ways to comprehend how the technological architecture of a blockchain-based healthcare system should be developed to handle such metadata from multiple sources [85], such as identity verification [67] and medicine procurement administration [136]. Although, in the midst of the ongoing COVID-19 pandemic, blockchain is anticipated to be a boon to the healthcare sector, especially by fostering the shared economy model. While the potential advantages of blockchain technology have benefited the pharmaceutical SC [74, 89], they have yet to be fully leveraged in the hospital management ecosystem due to inadequate technical infrastructure in many parts of the world [134]. This necessitates additional research into blockchain’s applicability for the healthcare SC, including data integration across hospital networks, electronic medical record management, hospital procurement management, and procurement protocols in the event of an emergency or disaster [134, 136].

### Future research agenda for manufacturing SC

The technical applicability of blockchain in the manufacturing SC has tremendous potential to positively contribute to the circular economy paradigm through environmental stewardship and the social equity agenda [33, 71]. Since manufacturing operations rely on global multi-echelon SCs, focal manufacturers from developed areas can encounter a digital disparity with some indigenous suppliers who are geographically dispersed in areas where technological development is sluggish [21, 55, 56]. This paves the way for further research into designing systemic mechanisms for mitigating such technical disparities in the interests of inter-organizational sustainability and greater regulatory compliance. Additionally, researchers have proposed the development of a blockchain-based shared platform that would allow manufacturers and their suppliers to participate in joint research and development (R&D) activities [77, 137]. For example, additive manufacturing methods (or 3D printing) can be more effective, have a lower environmental footprint than traditional (or subtractive) manufacturing processes, and provide greater agility in terms of product customization and reducing counterfeit product issues [22, 39, 137]. Given the high investment cost versus the potential advantages of additive manufacturing in a multi-echelon SC, future studies can explore and propose, using a case study methodology, the different financing and supporting mechanisms through which a focal manufacturer in a multi-echelon SC can assist their suppliers in implementing additive manufacturing practices. Such research opportunities can be expanded even further by including case-based procedural evidences from both developed and developing economies on risk sensitivity assessment and smart contracting.

### Future research agenda for infrastructure SC

The concept of smart cities is still in its infancy, and urban planning considerations of technological maturity and scalability using blockchain-enabled approaches make it a complex and rapidly-growing area of research [122, 123]. As a result, several major research opportunities can be pursued prior to mass deployment to solve metropolitan issues associated with smart city conceptualization, which lie in diverse areas of construction, built environment, energy, and transportation. Regardless of these diverse subsectors, blockchain-based solutions for the infrastructure SC face a common set of problems that require further research in application areas such as technical scalability [66, 73], data standardization [127], smart contract-based permission management [94], system interoperability [122], consensus reaching in vehicular networks [126], data security and privacy [131], and regulatory compliance management [13, 123].

## Conclusion

This systematic literature review has delivered on its goal to provide a state-of-the-art overview of high-quality research on blockchain for SSCM. Specifically, this review drew on evidence across sectors from both developed and developing countries, revealing a scarcity of studies in each sectoral domain. This review also highlighted the utility of blockchain for SSCM, and the nascent emergence of its study, thereby indicating that additional research is required to establish its technological significance and scale its potential. In this regard, this review encourages governments, policymakers, and all concerned stakeholders to prioritize actions to increase the technological penetration of blockchain in SC activities, with the end goal of enhancing the socioeconomic wellbeing of SC participants as well as preserving the environmental health of the planet. Noteworthily, this review makes clear that many opportunities avail for new researcher across sectors in the field, with ample room for conceptual development, situational reporting, and procedural contributions, among others. Indeed, sustainability in SC is a fast-growing trend with significant implications for economic and social development, and its deployment will lead to more sustainable and prosperous economies.

To this end, this review offers two major contributions. First, this review sheds light on the technical relevance of blockchain for SSCM across sectors, and provides generous suggestions to fertilize future research in the field. Second, this review reinforces the promise of blockchain to improve inter-organizational transparency among SC participants in the digital era, and hopefully, reduces skepticisms about incorporating blockchain into organizational plans or across the SC. Nonetheless, the insights in this review remains limited to that published in journals ranked “A*” or “A” or “B”, which only avail very recently (2018 onwards). Thus, periodic reviews of high-quality research should be pursued to provide updates on this nascent yet rapidly proliferating field.
